# Evaluation of metatranscriptomic sequencing protocols to obtain full-length RNA virus genomes from mammalian tissues

**DOI:** 10.1371/journal.pone.0324537

**Published:** 2025-05-30

**Authors:** Yiqiao Li, Mariana Polychronopoulou, Ine Boonen, Antonios Fikatas, Sophie Gryseels, Anne Laudisoit, Joelle Gouy de Bellocq, Bram Vrancken, Gkikas Magiorkinis, Philippe Lemey, Magda Bletsa

**Affiliations:** 1 Department of Microbiology, Immunology and Transplantation, KU Leuven, Rega Institute, KU Leuven, Leuven, Belgium; 2 Ruijin Hospital, Shanghai Jiao Tong University School of Medicine, Shanghai, PR China; 3 Department of Hygiene, Epidemiology and Medical Statistics, School of Medicine, National and Kapodistrian University of Athens, Athens, Greece; 4 Evolutionary Ecology group (EVECO), Department of Biology, University of Antwerp, Antwerp, Belgium; 5 EcoHealth Alliance, New York, New York, United States of America; 6 Institute of Vertebrate Biology, The Czech Academy of Sciences, Brno, Czech Republic; 7 Spatial Epidemiology Lab (SpELL), Université Libre de Bruxelles, Bruxelles, Belgium; 8 Bioinformatics and Applied Genomics, Department of Microbiology, Hellenic Pasteur Institute, Athens, Greece; Rani Lakshmi Bai Central Agricultural University, INDIA

## Abstract

High-throughput sequencing technologies have advanced RNA virus genomics, but recovering viral genomes from mammalian tissues remains challenging due to the predominance of host RNA. We evaluated two metatranscriptomic workflows to address these challenges. Our results demonstrate that the methods differed significantly in performance, with Method B achieving a 5-fold increase in RNA yield and improved RNA integrity over Method A. These differences resulted in the recovery of 4 complete hepacivirus genomes with Method B compared to fragmented or incomplete genomes with Method A. Additionally, Method B’s library preparation workflow, incorporating rRNA depletion, enhanced viral genome recovery by reducing host RNA background. Our novel approach integrates an optimized RNA purification protocol with a customized bioinformatics strategy for improved viral genome recovery. Overall, our findings highlight the critical role of optimized homogenization, RNA purification, and library preparation in metatranscriptomic workflows, facilitating the more effective RNA virus genome recovery from complex mammalian tissues.

## Introduction

Although viruses comprise the most abundant biological entities on Earth, it is estimated that only a tiny fraction - about 0.01% - of the virosphere has been discovered to date [1]. Expanding the knowledge of virus diversity has been a key focus in the post-genomic era, though our perspective on the global virome is strongly biased towards identifying causative agents of infectious diseases in humans or other economically important host species [2]. Among these agents, RNA viruses have garnered particular attention due to their significant role in epidemiology for causing a large fraction of infectious diseases and their grave threat to global public health [3,4].

Epidemiological studies, including novel pathogen discovery [5], variant identification [6], and comprehensive genome-wide evolutionary analysis [7], have greatly benefitted from obtaining the molecular information of agents, which becomes more accessible thanks to the advancement of the metagenomic next-generation sequencing (mNGS) technologies. This is conveniently achieved by extracting the total DNA and/or RNA, followed by preparing a library and shotgun sequencing [8]. Such untargeted approaches allow the unbiased detection of microbes and unlike traditional methods (e.g., PCR) that require prior knowledge of viral sequences, mNGS can detect known, novel, and divergent viruses without the need for specific primers [9]. By adopting different library preparation protocols and advanced techniques, such as rRNA depletion and low-input RNA purification protocols, we can now enhance the detection of low-abundance viral RNA, while improved bioinformatics tools enable accurate genome assembly and differentiation from host transcripts [10,11]. Moreover, the ability to sequence millions of reads in a single run ensures that even rare or scarce viral nucleic acids can be detected amidst a predominant background of host DNA or RNA. These features make metatranscriptomics highly effective for handling complex biological samples such as tissues, blood, and environmental samples, thus providing flexibility for studying RNA virus diversity, co-infections, and novel pathogen discovery in diverse environments [12].

Current metatranscriptomic workflows can face significant challenges when applied to mammalian tissues, potentially limiting their utility in viral surveillance and epidemiological studies [1]. This is primarily due to the overwhelming abundance of host RNA, which often obscures the detection of low-abundance viral RNA. Existing enrichment methods, such as rRNA depletion or poly(A) selection, are not always effective, particularly for non-polyadenylated or structurally diverse viral genomes [5]. Additionally, degraded RNA from tissue samples and biases in library preparation further hinder accurate detection. The high divergence of viral genomes and the reliance on incomplete reference databases complicate identification, while contaminants and off-target amplification introduce noise and false positives. These issues, coupled with logistical challenges in sample collection and processing, limit the sensitivity, specificity, and overall effectiveness of these workflows for viral surveillance and epidemiological studies [13].

The comparatively low abundance of RNA viruses is commonly attributed to their smaller genome size (up to 31,000 nucleotides) and lower number of genome copies compared to host genomes [14], which are argued to be caused by their high mutation rate (range between 10^-6^ and 10^-4^ s/n/c) and the lack of proofreading error correction systems [15,16]. With such high mutation rates, RNA viruses accumulate considerable numbers of insertions/deletions (indels), single-nucleotide variants (SNVs), and genomic reorganizations within their viral genomes that complicate genomic structures [17,18], thus, limiting the recovery of complete genomic information of an RNA virus without a proper metatranscriptomic workflow.

While input RNA load and integrity are varying by different sample types and storage conditions [19,20], the entire RNA extraction and purification process is crucial to ensure proper cell disruption for RNA release and to prevent RNA degradation and contamination. The library preparation is particularly important for the successful reconstruction of complete RNA virus genomes, involving proper selection of the library kit, appropriate fragmentation treatment and rRNA depletion steps [21]. However, while significant effort has been put for recovering complete genomes post-NGS using PCR or other targeted methods, fewer workflows have been optimized to obtain complete RNA virus genomes from mammalian tissues using metatranscriptomics.

In this study, we address a critical gap in viral genome recovery from mammalian tissues, with the goal of identifying the most efficient workflow for future applications in virology. To achieve this, we assessed the performance of two metatranscriptomic workflows that have been successfully applied to obtain whole genomes of RNA viruses [22,23]. The two workflows consist of different pretreatment methods for RNA isolation and different whole transcriptome sequencing protocols with enrichment strategies. In addition to this protocol assessment, we also provide a bioinformatic analysis pipeline for generating full-length RNA virus genomes from mammalian specimens. Our novelty lies in the combination of a specialized RNA purification protocol and a tailored bioinformatic approach to increase accuracy and efficiency of viral genome recovery from mammalian tissue samples.

## Materials and methods

### Sample collection

Kidney and spleen tissues for six rodent individuals were sourced from a large-scale screening study for hepacivirus detection [23]. For the present study, the selection of samples was guided by the availability of sufficient tissue material, which was necessary to perform the extensive testing required. As an additional parameter, we tried to include in our collection different sample types, different sampling locations, sampling dates and sampled rodent species for the comparison.

Samples have been collected as part of a collaborative effort between the University of Antwerp (Antwerp, Belgium) and the Institute of Vertebrate Biology (Brno, Czech Republic) to perform research on the molecular ecology of rodent populations in sub-Saharan Africa [24,25]. Small mammals were captured in Sherman live traps baited with a mixture of peanut butter and maize flour. In case of trapping success, individuals were euthanized by isoflurane inhalation. Spleen and kidney organs were collected and preserved in RNAlater (catalog #AM7020, ThermoFisher Scientific, Belgium) immediately after collection. RNAlater samples were kept at 4 °C for maximum six weeks prior to storage at -80 °C.

The six specimens used in this study, consists of one specimen collected in 2010 from the Democratic Republic of Congo, three specimens collected in 2011 from Mozambique and two samples collected in 2013 from Tanzania. In Table 1 we provide further details for these specimens, including the rodent species and family information, the sampling country and year, geographical coordinates of the sampling sites and the type of tissue material collected.

**Table 1:** Information on the rodent specimens used in this study.

sample	species	familiy	country	year	latitude	longitude	material
**CRT125**	*Lophuromys dudui*	Muridae	Congo	2010	0.82099	24.27678	kidney
MOZ002	*Micaelamys namaquensis*	Muridae	Mozambique	2011	-19.7105	33.0069	kidney
MOZ091	*Lophuromys machangui*	Muridae	Mozambique	2011	-16.3057	36.4241	spleen
**MOZ135**	*Lophuromys machangui*	Muridae	Mozambique	2011	-16.3086	36.4245	spleen
TA106	*Lophuromys stanleyi*	Muridae	Tanzania	2013	-1.031	31.572	kidney
TA531	*Lophuromys machangui*	Muridae	Tanzania	2013	-9.04075	33.5732	kidney

### RNA purification assays

For all six samples that we focus on here, two methods of sample homogenization and pre-RNA purification treatment (from now on referred to as pretreatment) were investigated, both followed by the same commercial RNA extraction protocol.

**Fig 1:**
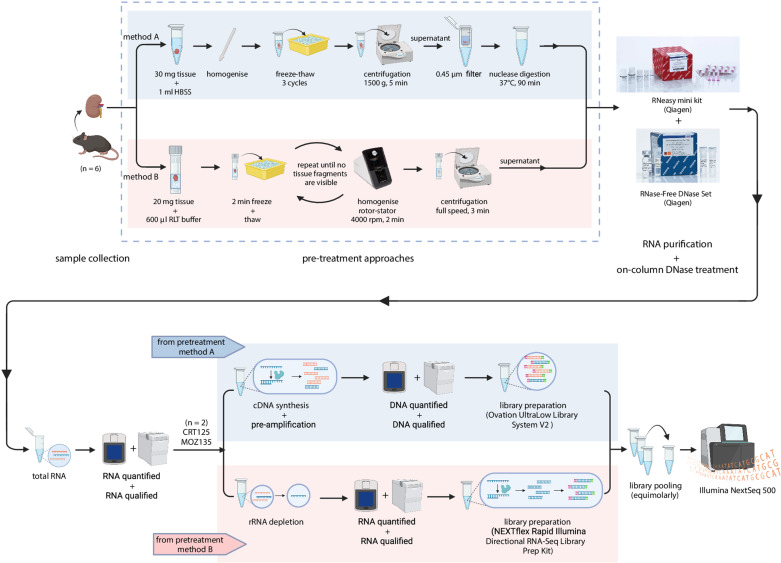
Schematic representation of the experimental design used in this study. Top panel: Two different pretreatment approaches were tested on six tissue specimens (kidney and spleen) from rodents. The steps of method A are demonstrated in blue color, while the steps of method B are shown in pink color. Bottom panel: each pretreatment method was followed by a specific metatranscriptomic workflow so as to prepare sequencing libraries rich in RNA metagenomes. The coloring scheme follows the respective pretreatment method.

In pretreatment method A (Fig 1, marked in blue color), samples were treated according to the viral enrichment protocol S3, described in [26]. In particular, a piece of rodent spleen or kidney (about 30 mg) was immersed in 1 ml of cold 1X Hank’s Balanced Salt Solution (catalog #14175053, Gibco™, ThermoFisher Scientific, Belgium) and homogenized with a micropestle. Upon homogenization with a micropestle, samples were subjected to three dry ice freeze-thaw cycles until cells were disrupted. This was followed by a centrifugation step for 5 min at 1,500g at 4°C to collect pellet nuclei and large cellular aggregates. The supernatant was filtered with a 0.45 μm membrane filter (catalog #HPWP01300, Millipore, Merck, Germany) and nuclease digestion was performed using 25U RNase One Ribonuclease (catalog #M4265, Promega, the Netherlands) and 30U of Benzonase Nuclease (catalog #E1014-25KU, Millipore, Merck, Germany) at 37°C for 90 min. Finally, RNA purification was performed using the RNeasy mini kit (catalog #74104, Qiagen, Belgium) according to the manufacturer’s instructions with an intermediate step of on-column DNase treatment with the RNase-Free DNase Set (catalog #79254, Qiagen, Belgium) to remove any residual DNA prior to RNA purification. Elution was performed twice by collecting and re-loading the same eluate on the filter column.

In pretreatment method B (Fig 1, marked in pink color), optimizations have been made to the homogenization recommendations of the RNeasy mini kit (catalog #74104, Qiagen, Belgium) followed by novel implementations of the freeze-thaw cycles to ensure a more efficient disruption and homogenization of the tissue material. In more detail, a piece of RNAlater stabilized spleen or kidney (maximum amount of 20 mg) was added to 600 μl RLT buffer into 2.8 mm ceramic beads lysate tubes (catalog #432-0141, Precellys, Bertin Instruments, VWR, Belgium). Tubes were then placed on dry ice for 2 min and immediately after thawing, tissues were disrupted using a rotor-stator homogeniser (catalog #432-0274, Minilys, Bertin Instruments, VWR, Belgium) at a medium speed of 4,000 rpm for 2 min. A second freeze-thaw cycle was performed followed by homogenisation until no tissue fragments were visible. The lysate was centrifuged for 3 min at full speed and the supernatant was used for RNA purification using an optimized version of the RNeasy mini kit (catalog #74104, Qiagen, Belgium). Prior to the washing steps, we performed an on-column DNase treatment with the RNase-Free DNase Set (catalog #79254, Qiagen, Belgium). Finally, in order to increase the yield of viral RNA during purification, we used the flow-through from the first elution to re-elute the column. The RNA purification protocol described in this section is published on protocols.io (https://dx.doi.org/10.17504/protocols.io.8ufhwtn).

### Metatrascriptomic library preparation assays for virus recovery

Purified RNA from both methods was quantified using the RNA Quantifluor System (catalog #E3310, Promega, the Netherlands) and RNA profiles were assessed with an Agilent RNA 6000 Nano kit on a Bioanalyzer 2100 (catalog #5067-1511, Agilent Technologies, Germany). RNA purified based on both protocols applied to specimens CRT125 and MOZ135 (see Table 1) were selected for further molecular investigation. As shown in Fig 1, two different workflows of pre-amplification and library preparation were tested for each specimen, both designed to enrich viral RNA recovery.

In the first workflow, the total RNA of the two specimens, purified through pretreatment method A, was subjected to cDNA synthesis and enriched by pre-amplification using the Ovation RNA-Seq System V2 (catalog #M01206v9, Tecan, Switzerland) (Fig 1, marked in blue color). The products of pre-amplification were quantified with the dsDNA Quantifluor System (catalog #E2670, Promega, the Netherlands) and qualified with the Agilent High Sensitivity DNA kit (catalog #5067-4626, Agilent Technologies, Germany). Sequencing libraries were constructed with the Ovation UltraLow Library System V2 (catalog #M01379v5, Tecan, Switzerland) without the fragmentation step, since the input DNA fragment sizes were relatively short. Libraries were pooled in an equimolar fashion and paired-end sequenced on an Illumina NextSeq 500 platform at Viroscan3D (Lyon, France) [22]. This library prep assay described in this subsection is published on protocols.io (https://dx.doi.org/10.17504/protocols.io.14egn6w96l5d/v1).

In the second workflow, the total RNA of the two specimens, purified through pretreatment method B, was subjected to a ribosomal RNA (rRNA) depletion step using the Ribo-Zero rRNA Depletion Kit (Human/Mouse/Rat) (catalog #20040526, Illumina, the Netherlands) on an input sample of 1 μg (Fig 1, marked in pink color). Upon depletion, RNA was quantified again using the RNA Quantifluor System (catalog #E3310, Promega, the Netherlands) and cDNA was generated, followed by library construction using the NEXTflex Rapid Directional RNA-Seq Library Prep Kit (catalog #NOVA-5198-01, Revvity, USA) without fragmentation. Finally, sequencing libraries were equimolarly combined into a pool and paired-end sequencing was performed on an Illumina NextSeq 500 platform at Viroscan3D (Lyon, France) [23]. The various steps of the library prep assay described in this subsection are published as two different protocols on protocols.io (https://dx.doi.org/10.17504/protocols.io.q26g74843gwz/v1 and https://dx.doi.org/10.17504/protocols.io.kqdg3p6pel25/v1).

### Viral genome reconstruction

**Fig 2:**
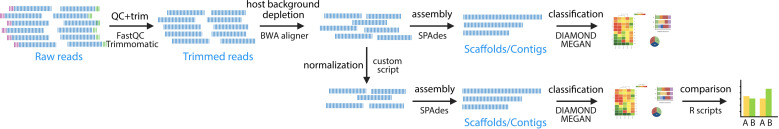
Simplified overview of our bioinformatic analysis pipeline for RNA virus discovery. Raw reads were quality controlled and trimmed by Trimmomatic. Host background reads were depleted by aligning to rodent reference genomes using BWA aligner. The complete host-depleted dataset was used for *de novo* assembly to generate RNA virus genomes. A normalized dataset was subjected to *de novo* assembly for performing comparisons between the different samples.

All sequencing outputs were analyzed using our metatranscriptomic analysis pipeline summarized in Fig 2 and consisting of the following steps. Adapter content and low-quality bases were removed using Trimmomatic v0.39 [27] with a PHRED score ≥ 30 and a minimum read length of 50 bp. To deplete host background reads, trimmed reads were mapped against three closely related host species with BWA v0.7.17-r1188 [28]. The following rodent reference genomes were used: *Acomys russatus* GCF_903995435.1, *Mastomys natalensis* GCA_019843795.1 and *Rattus norvegicus* GCF_015227675.2. At this point two datasets were defined: i) the complete dataset, which included all the unmapped reads after the digital host depletion, and ii) the normalized dataset, which was prepared to allow for sample-to-sample comparisons. Normalization across barcoded samples was performed by randomly selecting a subset of paired-end reads per metagenome, with the size fixed to the sample with the fewest number of host-depleted reads. For each dataset, *de novo* assembly was performed using both SPAdes v3.15.5 and metaSPAdes v3.15.5 with default parameters [29]. Evaluation and comparison of the metatranscriptome assemblies was conducted using metaQuast v5.2.0 [30]. Viral contigs were identified upon comparison of the assembled contigs against the NCBI Riboviria RefSeq database using DIAMOND v0.9.19 (BLASTX search) with e-value cutoff ≤ 1E-5 and in *-sensitive* mode [31] and by blast similarity search against the NCBI non-redundant protein (NR) database to ensure that our viral-like contigs were of exogenous origin.

### Taxonomic classification and virus abundance metrics

Taxonomic assignment of the viral contigs was conducted using MEGAN6 v6.21.7 [32] and the resulting output was visualized with KronaTools v2.8.1 [33] for each Riboviria taxonomic rank. Contig abundance was determined by iteratively mapping the normalized reads against each viral contig using BWA v0.7.17- r1188 [28]. Depth and breadth of coverage were calculated using Samtools v1.6 [34] and consensus sequences were called for the most abundant RNA virus contigs for only the normalized dataset. Information on the number of raw, trimmed, host-depleted, normalized viral sequence reads is detailed in Table 2. All the raw data from this study have been deposited in the NCBI Sequence Read Archive repository under BioProject accession number PRJNA720117 (SRA accession numbers: SRR26210002 – SRR26210005).

**Table 2:** Sequencing results and statistics obtained from methods A and B for samples CRT125 and MOZ135. (reads* indicates paired end read data).

Method	Sample	Total reads*	reads mapping to host	% reads mapping to host	reads mapping to reference	% reads mapping to reference	Mean depth (x)	Coverage %	Reference genome	Reference size (nts)
A	CRT125	7,675,533	7,185,009	93.61	2	0.003	0.04	3.07	CRT125-B	10,198
					68		1.49	11.19	CRT125-C	9,556
					2		0.06	6.45	CRT125-D	7,378
	MOZ135	25,652,787	20,043,989	78.14	236,454	1.26	3,295.07	100	MMLV1	17,940
					87,316		1,267.65	100	MMLV2	17,220
B	CRT125	38,253,680	4,259,910	11.14	31,176	0.22	618.28	100	CRT125-A	10,589
					10,741		221.18	99.86	CRT125-B	10,198
					39,020		857.49	100	CRT125-C	9,556
					4,482		127.57	100	CRT125-D	7,378
	MOZ135	27,237,820	1,684,561	6.18	–	–	–	–	MMLV1	17,940
					–		–	–	MMLV2	17,220

### Ethics statement

This study used specimens obtained from previous studies that were conducted in accordance with the ethical guidelines and regulations of the University of Antwerp Ethical Committee for Animal Experimentation (2011-52) and complied with regulations of the Research Policy of Sokoine University of Agriculture as stipulated in the Code of Conduct for Research Ethics. No new data collection involving animals was conducted in the present study. Therefore, no additional ethics approval was required for this work.

## Results and discussion

### Pretreatment methods impact the total RNA quantity and quality

**Fig 3:**
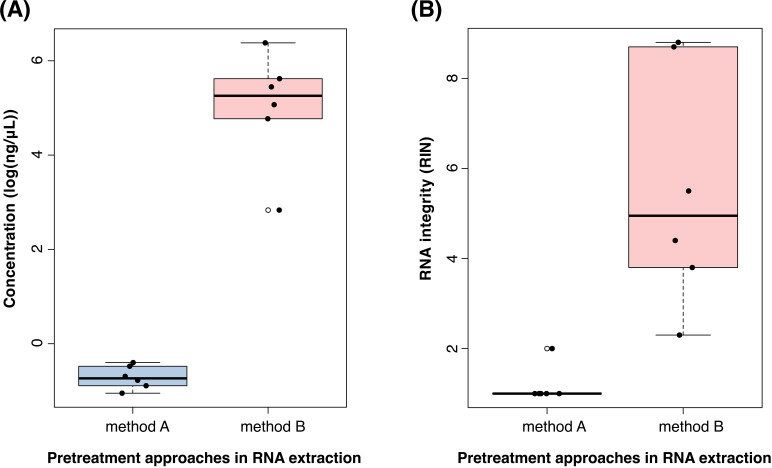
Box-and-Whisker plots of the total RNA quantity and quality between two pretreatment methods. **(A)** Comparison of the natural logarithm of the total RNA concentration between pretreatment methods A and B. **(B)** Comparison of the RNA Integrity Number (RIN) between pretreatment methods A and B. Each tested protocol is represented by a boxplot and the results from the six processed samples are indicated with circles. For each method, the median value (horizontal black line) and the interquartile range (colored box) are shown, as well as the 25th and 75th quartiles (lower and upper box boundaries, respectively) and the minimum and maximum values (whiskers).

In the present work, we subjected six rodent tissue specimens to two different pretreatment protocols followed by RNA purification with the same commercial kit. To compare the outcome of the two methods, we measured the RNA concentration after the final elution step. For pretreatment method A, the yield of the total RNA ranged from 0.35 ng/μL to 0.67 ng/μL with a median value of 0.48 ng/μL (S1 Table in S1 File). For method B the total RNA yield varied between 17 ng/μL and 590 ng/μL with a median value of 196 ng/μL (S1 Table in S1 File). As demonstrated in Fig 3A, the quantity of RNA obtained from the pretreatment protocol in method B was significantly higher compared to that of method A, albeit with apparently more variation for method B.

While RNA quantity is crucial to measure prior to preparing a metatranscriptomic library, the quality of RNA is equally important to assess. As described in the Methods section, we also evaluated the two pretreatment methods by characterizing integrity of the RNA fragments present in each sample. Based on the RNA Integrity Number (RIN), which is an objective measure of RNA quality ranging from 1 (totally degraded) to 10 (least degraded), samples treated with method A resulted in RIN values 1 and 2 with a median of 1 (S1 Table in S1 File). Pretreatment method A therefore consistently produced highly degraded RNA and, thus, offered little reassurance for a successful outcome of the downstream experiments. Contrary to the poor RNA quality of samples treated with method A, when the same samples were processed with method B, they resulted in RIN values ranging from 2.3 to 8.8 with a median value of 4.95 (S1 Table in S1 File). The fact that method B outperformed method A is further illustrated in Fig 3B, which plots the distribution of the results from the RNA quality assessment for all six tested samples.

This outspoken difference in RNA concentration and integrity between the two methods can most probably be attributed to the low-speed centrifugation, syringe-based filtration and nuclease digestion, which were performed in pretreatment method A but not in method B. Although these three steps are routinely applied for viral enrichment [35], in our experiment we observed that they did not substantially increase the RNA yield and viral abundance in any of the tested samples.

Besides the low RNA concentration, we suspect that nuclease digestion prior to RNA purification was most probably the step that had the greatest effect on the severe degradation of RNA. The on-column DNase digestion likely offers a substantial enrichment advantage since it specifically removes the DNA fragments present in our samples. However, this step has been performed after both pretreatment methods and we cannot assess the impact of this step on the overall metatranscriptomic workflow.

Based on previous literature [26] we were expecting that the freeze-thaw cycles in pretreatment method A would facilitate the lysis of the host cells, thus releasing additional nucleic acids and a larger number of viral particles. However, the RNA yield from samples subjected to the three freeze-thaw cycles was consistently low. In addition, most of the published protocols for improved recovery of virus genomes suggest that sample homogenization should be mild -ideally carried out using a pestle- and centrifugation should preferably be performed at a low speed [26,36]. In our experience, micropestle homogenization can be cumbersome for shearing fibrous tissues such as spleen and often results in less optimal homogenization, since cellular aggregates are still present in the samples compared to the rotor-stator homogenization processes with the aid of ceramic beads. While we agree that low-speed centrifugation most likely minimizes RNA shearing, we noticed that a centrifugation step of the lysate at full speed was essential prior to loading the supernatant into the RNA purification columns.

Overall, the optimizations introduced in pretreatment method B provided improved results for the successful recovery of RNA in the mammalian tissue types we investigated here. This is because pretreatment method B had superior performance with respect to both the RNA yield obtained and the quality of purified RNA. Higher RNA yield is essential for successful viral genome reconstruction as it ensures sufficient template material and minimizes the risk of introducing sequencing artifacts or errors due to overamplification [37]. In addition, poor-quality RNA, characterized by degradation or contamination, can lead to incomplete or biased amplification, resulting in gaps in genome coverage or inaccurate sequence representation [38]. Therefore, better RNA quality directly correlates with higher fidelity in genome reconstruction. This reliability is crucial for the accuracy of downstream applications like estimation of viral genetic diversity, recombination analysis and phylogenetics.

### Library preparation methods might have a strong effect on Illumina sequencing output

**Fig 4:**
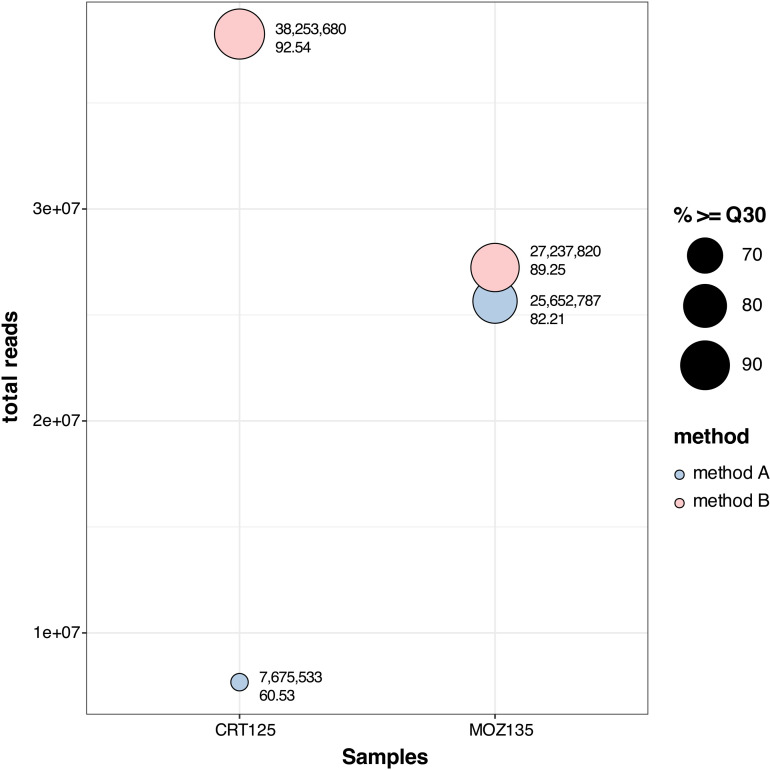
Sequencing output from CRT125 and MOZ135 using the two metatranscriptomic workflows. The x-axis represents the two samples tested and the y-axis reports the total number of reads obtained from paired-end sequencing. Colored circles denote the results per sample and per method applied, with the circle size being proportional to the percentage of reads with accuracy ≥Q30, ranging from 70% to 90%. Next to each circle, we show the exact number of total reads obtained and the exact proportion of reads belonging to the ≥ Q30 threshold. Blue circles correspond to the sequencing output of workflow A and pink to the output of workflow B.

The samples for which we had plenty of initial material for testing purposes, specimens CRT125 and MOZ135, were selected and processed with the two different metatranscriptomic protocols (Fig 1). As a result of Illumina sequencing of both samples, a total number of 33,328,320 paired-end reads was produced from the library prep method A with 71.37% of those reads having a Phred quality score of 30 or higher (≥Q30). From library prep method B, we obtained 65,491,500 paired-end reads for both samples (S1 Table in S1 File) with over 90.9% of those having an accuracy of ≥Q30 (Fig 4).

Already from the Illumina output, we observe that the second workflow yielded almost twice the total number of reads, and that the proportion of reads showing 99.9% accuracy (≥Q30) was much higher in method B compared to method A. We cannot directly attribute the increased number of reads generated with workflow B to the library prep method used, since it is highly dependent on the quantity and quality of the RNA input. However, we can assess the accuracy and quality of the generated read data and based on our results we notice a dramatic increase in the proportion of reads having a quality score ≥Q30. Whether this improvement derives from the library preparation kit selected or the combined use of pretreatment method B and rRNA depletion is something that requires further investigation.

Due to the unequal number of reads produced from the two sequencing runs, we ensured comparability between outputs from the two different workflows by normalizing the number of reads per sample and per sequencing run. *De novo* assembly was performed on both the original and the normalized datasets using two different algorithms (SPAdes and metaSPAdes). Contrary to typical expectations for complex metatranscriptomic datasets, quality assessment of the generated assemblies resulted in SPAdes producing more contiguous assemblies with higher viral genome completeness compared to metaSPAdes (S2 Table in S1 File). Contigs assembled from the normalized dataset and the SPAdes algorithm were used for viral diversity investigations and comparisons.

### Different pretreatment and metatranscriptomic assays uncover distinct viral diversity

**Fig 5:**
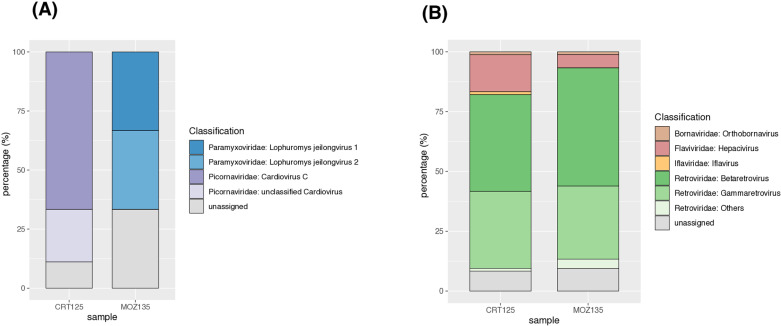
Composition of ribovirus abundance and diversity in normalized datasets. The abundance of contigs of each riboviral families obtained through method A (A) and method B (B) are shown with stacked plots.

The assembled contigs from the normalized datasets were compared against the NCBI Riboviria RefSeq database and their abundance and diversity within each viral family were visually represented using stacked plots and Krona plots, respectively (Fig 5).

The stacked plots reveal the different compositions of RNA viruses (*Riboviria*) in both samples obtained from the different methodological approaches. The application of method A (Fig 5A) primarily resulted in members from *Picornaviridae* in sample CRT125. In contrast, sample MOZ135 shows the identification of two species within *Paramyxoviridae*, both at similar proportions, but are obviously lower (>50% in total) compared to the levels of *Picornaviridae* in CRT125 (>75%). When using method B (Fig 5B), both samples show a competitive proportion of unassigned contigs and predominantly contain contigs belonging to the *Retroviridae* family.

Based on the specifics of each viral family, for CRT125, when using method A, only 9 contigs were identified as RNA viruses with one of them (11%) being unassigned. The remaining 8 contigs were all associated with the *Cardiovirus* genus of the family *Picornaviridae* (S1A Fig in S1 File). However, for sample CRT125 processed with method B, a total of 84 contigs were ribovirus-related, while 7 contigs (8%) remained unassigned. Among the 77 riboviral contigs, the majority (*n = *62 contigs) belong to the *Retroviridae* family, mainly *Betaretrovirus* and *Gammaretrovirus* genus, while the remaining contigs (*n = *15) are classified within the orthornaviran taxa, particularly in the *Hepacivirus* genus (*n = *13 contigs) (S1B Fig in S1 File).

For MOZ135 processed with method A, 57 contigs were classified as ribovirus-related while 18 contigs (32%) were not assigned to any known taxonomy. Among the 39 well-assigned contigs, 38 were classified as Jeilongvirus, with only one falling into another ribovirus group (Fig S1C in S1 File). In contrast, for sample MOZ135 processed with method B, a total number of 180 contigs were classified as RNA viruses and 17 contigs (9%) remained unassigned. Out of the 163 assigned contigs, 151 of them were classified as *Retroviridae*, while the remaining 12 belonged to the Orthornavirae kingdom, with the majority (n = 10 contigs) assigned to the *Hepacivirus* genus (S1D Fig in S1 File).

Overall, an increased number of RNA virus contigs were obtained from both CRT125 and MOZ135 specimens using method B (*n = *264 contigs in total) compared to those obtained using method A (*n = *66 contigs in total). In addition to that, contigs from method B exhibit a slightly higher riboviral diversity and they contain a somewhat smaller proportion of unassigned riboviral contigs (8% and 9%, respectively, for CRT125 and MOZ135) compared to the unassigned proportion of contigs obtained with method A (11% and 32%, respectively, for CRT125 and MOZ135). Finally, comparison of the viral populations between the two samples reveals a discrepancy in virus diversity obtained from the two methods. For instance, application of method A, resulted in the identification of *Jeilongvirus* and *Cardiovirus* genera, which comprise the largest proportion of virus diversity, while they are completely absent in the samples treated with method B. Conversely, hepaciviruses were consistently identified in both samples among the assigned non-retrovirus *Riboviria* hits only when we applied the second method.

### Complete genomes of paramyxoviruses and hepaciviruses assembled using the different methods

In previous studies we have successfully detected hepaciviruses in CRT125 and MOZ135 samples [23] as well as paramyxoviruses in specimen MOZ135 [22]. Therefore, we aimed to generate full genomic insights from those previously detected RNA viruses with two alternative methodologies and compare the recovery success of each approach on the different viruses.

As summarized in Table 2, method A recovered only tiny genomic fragments of hepaciviruses from specimen CRT125 and failed to detect any hepaciviruses from specimen MOZ135. The coverage of those four hepacivirus genomes, previously named as CRT125-A – CRT125-D, varied between 3–11%. Only 0.003% of the reads mapped to those four hepaciviruses with a mean sequencing depth ranging from 0.04x to 3.17x (Table 2). Contrary to the very small proportion of hepacivirus-specific reads, about 93% of the reads mapped to the host genome, indicating a major shortcoming of this methodological approach to selectively recover RNA virus genome fragments in a background of host material.

While hepaciviruses were not fully sequenced with the first workflow, this was not the case for paramyxoviruses. Specifically, with method A we managed to reconstruct two complete genomes of paramyxoviruses with 1.26% of the reads mapping to both Mount Mabu Lophuromys Virus 1 (MMLV1) and Mount Mabu Lophuromys Virus 2 (MMLV2). Although the mean sequencing depth of those paramyxovirus genomes ranged from 1,267x for MMLV2 to 3,295x for MMVL1, the host background was relatively high with 78.14% of the initial reads mapping to the host genome (Table 2).

On the other hand, method B recovered almost complete genomes of the four hepaciviruses with 0.22% of the reads mapping to them. The proportion of host-specific reads was dramatically lower compared to method A and accounted for 11.14% of the total reads. The mean sequencing depth for each hepacivirus varied from 127x for CRT125-D to 857x for CRT125-C. Finally, we did not detect any reads mapping to the paramyxovirus genomes in the MOZ135 specimen with this method. However, the proportion of reads mapping to the host genome for this sample was only about 6% for the second workflow.

In summary, complete paramyxovirus genomes were recovered only from the first workflow, which included cDNA synthesis using oligo-dT and random hexamers, followed by a Single Primer Isothermal Amplification (SPIA) step. Paramyxoviruses, when transcribed, form 3’ polyadenylated (A) mRNAs, which are later reverse transcribed using an oligo-dT primer. Therefore, in method A we have preferentially enriched for host transcripts and RNA viruses possessing a poly-A tail, such as viruses from the *Paramyxoviridae* family. In method B, we have selectively removed any rRNA by employing DNA probes complementary to rodent rRNA to hybridize and subsequently digest those rRNA host nucleic acids. In line with previous studies, we observed that rRNA depletion not only enhanced the recovery of diverse RNA viruses from tissues with high host background, but also increased the sensitivity of virus detection in ultra-low RNA input samples [39].

The results from our comparative analysis of the SPAdes and metaSPAdes *de novo* assembly algorithms suggest that our dataset may represent a less diverse or more uniform viral community, better aligned with the assumptions of the SPAdes algorithm. Comparative assessment using metrics such as N50, L50, total contig length, length of the largest contig support the superior performance of SPAdes in this context. These findings underscore the importance of tailoring assembly strategies to dataset characteristics, as standard metagenomic/metatranscriptomic assemblers may not always yield optimal results for viral transcriptomes.

Although this is a limited study, our results indicate that method B could be a preferred approach for full genome sequencing of hepaciviruses because the high sequencing depth may still allow recovering viral RNA in low copy numbers. While we acknowledge that a larger sample size would strengthen the statistical power of our findings, the current study was designed to demonstrate proof-of-concept of a methodological protocol within these constraints. Confronted with the challenge of restricted sample size, we took several steps to ensure the validity and reliability of our findings. These include stringent quality control measures during RNA extraction and sequencing, the use of a negative control during all wet-lab steps and the application of a robust bioinformatics pipeline to minimize potential biases. However, caution is needed when interpreting our findings because of the limited number of samples compared in the metatranscriptomic workflow and because we did not include multiple replicates of these samples in the specific workflows. We therefore lack information on what variability in outcome can be expected for each workflow and further research is needed to characterize this with larger sample sizes.

## Conclusion

Our findings underscore the inherent challenges in designing efficient viral metatranscriptomic assays for the recovery of RNA virus genomes from mammalian tissue samples. The outcome of these methods is influenced by multiple factors, including the specific pathogens targeted, the abundance of viral RNA, sample homogenization techniques, the choice of RNA purification kits, the primers used for cDNA synthesis, the inclusion of rRNA depletion steps, the library preparation protocol, the sequencing platform, and the bioinformatic analyses employed. Additionally, resource constraints play a significant role in determining the feasibility and scalability of these approaches.

To improve the success of viral metatranscriptomic workflows, our proof-of-concept approach combines a custom RNA purification protocol with a tailored bioinformatics strategy, addressing challenges such as pathogen targeting, RNA abundance, and resource constraints for enhanced viral genome recovery from mammalian tissues. Future research should focus on standardizing protocols and systematically assessing the impact of each variable on the recovery and accuracy of viral genomes. Moreover, efforts to develop cost-effective, all-in-one solutions that integrate optimized RNA extraction, library preparation, and sequencing steps could address current limitations. Expanding reference databases and improving bioinformatic pipelines will also be crucial to enhancing the sensitivity and specificity of viral genome identification.

## Supporting information

S1 File Supplementary materials: Table S1: Results of RNA purification and sequencing. Table S2: Results of the comparison between SPAdes and metaSPAdes algorithms for the *de novo* assembly of the reads from the normalized subsets. Figure S1: Composition of ribovirus diversity and abundance in normalized datasets.
(PDF)

S2 File High-quality RNA purification with on-column DNase treatment from tissue specimens. Also available on protocols.io: https://dx.doi.org/10.17504/protocols.io.8ufhwtn
(PDF)

S3 File NGS library preparation using Ovation RNA-Seq System V2 (M01206v9) and Ovation Ultralow System V2 (M01437 v2) for animal tissue samples. Also available on protocols.io: https://dx.doi.org/10.17504/protocols.io.14egn6w96l5d/v1
(PDF)

S4 File NGS library preparation using NEXTFLEX Rapid Directional RNAseq kit (NOVA-5138-08) for animal tissue samples. Also available on protocols.io: https://dx.doi.org/10.17504/protocols.io.q26g74843gwz/v1
(PDF)

S5 File NGS workflow with rRNA depletion for viral RNA sequencing from animal tissue specimens. Also available on protocols.io: https://dx.doi.org/10.17504/protocols.io.kqdg3p6pel25/v1
(PDF)

## References

[1] Cobbin JC, Charon J, Harvey E, Holmes EC, Mahar JE. Current challenges to virus discovery by meta-transcriptomics. Curr Opin Virol. 2021;51:48–55. doi: 10.1016/j.coviro.2021.09.007 34592710

[2] Zhang Y-Z, Chen Y-M, Wang W, Qin X-C, Holmes EC. Expanding the RNA Virosphere by Unbiased Metagenomics. Annu Rev Virol. 2019;6(1):119–39. doi: 10.1146/annurev-virology-092818-015851 31100994

[3] Jones KE, Patel NG, Levy MA, Storeygard A, Balk D, Gittleman JL, et al. Global trends in emerging infectious diseases. Nature. 2008;451(7181):990–3. doi: 10.1038/nature06536 18288193 PMC5960580

[4] J Woolhouse ME, Adair K, Brierley L. RNA Viruses: A Case Study of the Biology of Emerging Infectious Diseases. Microbiol Spectr. 2013;1(1):10.1128/microbiolspec.OH-0001–2012. doi: 10.1128/microbiolspec.OH-0001-2012 26184815 PMC6157708

[5] Vanmechelen B, Zisi Z, Gryseels S, Goüy de Bellocq J, Vrancken B, Lemey P, et al. Phylogenomic Characterization of Lopma Virus and Praja Virus, Two Novel Rodent-Borne Arteriviruses. Viruses. 2021;13(9):1842. doi: 10.3390/v13091842 34578423 PMC8473226

[6] Kutkat O, Gomaa M, Moatasim Y, El Taweel A, Kamel MN, El Sayes M, et al. Highly pathogenic avian influenza virus H5N1 clade 2.3.4.4b in wild rats in Egypt during 2023. Emerg Microbes Infect. 2024;13(1):2396874. doi: 10.1080/22221751.2024.2396874 39193629 PMC11382695

[7] Tsui JL-H, McCrone JT, Lambert B, Bajaj S, Inward RPD, Bosetti P, et al. Genomic assessment of invasion dynamics of SARS-CoV-2 Omicron BA.1. Science. 2023;381(6655):336–43. doi: 10.1126/science.adg6605 37471538 PMC10866301

[8] Zhang L, Chen F, Zeng Z, Xu M, Sun F, Yang L, et al. Advances in Metagenomics and Its Application in Environmental Microorganisms. Front Microbiol. 2021;12:766364. doi: 10.3389/fmicb.2021.766364 34975791 PMC8719654

[9] Ko KKK, Chng KR, Nagarajan N. Metagenomics-enabled microbial surveillance. Nat Microbiol. 2022;7(4):486–96. doi: 10.1038/s41564-022-01089-w 35365786

[10] DeLong EF. The microbial ocean from genomes to biomes. Nature. 2009;459(7244):200–6. doi: 10.1038/nature08059 19444206

[11] Poretsky RS, Hewson I, Sun S, Allen AE, Zehr JP, Moran MA. Comparative day/night metatranscriptomic analysis of microbial communities in the North Pacific subtropical gyre. Environ Microbiol. 2009;11(6):1358–75. doi: 10.1111/j.1462-2920.2008.01863.x 19207571

[12] Shakya M, Lo C-C, Chain PSG. Advances and Challenges in Metatranscriptomic Analysis. Front Genet. 2019;10:904. doi: 10.3389/fgene.2019.00904 31608125 PMC6774269

[13] Chiu CY, Miller SA. Clinical metagenomics. Nat Rev Genet. 2019;20(6):341–55. doi: 10.1038/s41576-019-0113-7 30918369 PMC6858796

[14] Chaitanya KV. Structure and Organization of Virus Genomes. Genome and Genomics. 2019;1–30.

[15] Belshaw R, Pybus OG, Rambaut A. The evolution of genome compression and genomic novelty in RNA viruses. Genome Res. 2007;17(10):1496–504. doi: 10.1101/gr.6305707 17785537 PMC1987338

[16] Peck KM, Lauring AS. Complexities of Viral Mutation Rates. J Virol. 2018;92(14):e01031-17. doi: 10.1128/JVI.01031-17 29720522 PMC6026756

[17] Aguilar Rangel M, Dolan PT, Taguwa S, Xiao Y, Andino R, Frydman J. High-resolution mapping reveals the mechanism and contribution of genome insertions and deletions to RNA virus evolution. Proc Natl Acad Sci USA. 2023;120(31). doi: 10.1073/pnas.2304667120 PMC10400975 37487061

[18] Elena SF. The role of indels in evolution and pathogenicity of RNA viruses. Proc Natl Acad Sci U S A. 2023;120(33):e2310785120. doi: 10.1073/pnas.2310785120 37531375 PMC10433266

[19] Relova D, Rios L, Acevedo AM, Coronado L, Perera CL, Pérez LJ. Impact of RNA Degradation on Viral Diagnosis: An Understated but Essential Step for the Successful Establishment of a Diagnosis Network. Vet Sci. 2018;5(1):19. doi: 10.3390/vetsci5010019 29415432 PMC5876574

[20] Poulsen CS, Kaas RS, Aarestrup FM, Pamp SJ. Standard Sample Storage Conditions Have an Impact on Inferred Microbiome Composition and Antimicrobial Resistance Patterns. Microbiol Spectr. 2021;9(2):e0138721. doi: 10.1128/Spectrum.01387-21 34612701 PMC8510183

[21] Head SR, Komori HK, LaMere SA, Whisenant T, Van Nieuwerburgh F, Salomon DR, et al. Library construction for next-generation sequencing: overviews and challenges. Biotechniques. 2014;56(2):61–4, 66, 68, passim. doi: 10.2144/000114133 24502796 PMC4351865

[22] Vanmechelen B, Bletsa M, Laenen L, Lopes AR, Vergote V, Beller L, et al. Discovery and genome characterization of three new Jeilongviruses, a lineage of paramyxoviruses characterized by their unique membrane proteins. BMC Genomics. 2018;19(1):617. doi: 10.1186/s12864-018-4995-0 30115009 PMC6097224

[23] Bletsa M, Vrancken B, Gryseels S, Boonen I, Fikatas A, Li Y, et al. Molecular detection and genomic characterization of diverse hepaciviruses in African rodents. Virus Evol. 2021;7(1):veab036. doi: 10.1093/ve/veab036 34221451 PMC8242229

[24] Gryseels S, Goüy de Bellocq J, Makundi R, Vanmechelen K, Broeckhove J, Mazoch V, et al. Genetic distinction between contiguous urban and rural multimammate mice in Tanzania despite gene flow. J Evol Biol. 2016;29(10):1952–67. doi: 10.1111/jeb.12919 27306876

[25] Gryseels S, Baird SJE, Borremans B, Makundi R, Leirs H, Goüy de Bellocq J. When Viruses Don’t Go Viral: The Importance of Host Phylogeographic Structure in the Spatial Spread of Arenaviruses. PLoS Pathog. 2017;13(1):e1006073. doi: 10.1371/journal.ppat.1006073 28076397 PMC5226678

[26] Dupinay T, Pounder KC, Ayral F, Laaberki M-H, Marston DA, Lacôte S, et al. Detection and genetic characterization of Seoul virus from commensal brown rats in France. Virol J. 2014;11:32. doi: 10.1186/1743-422X-11-32 24555484 PMC3944734

[27] Bolger AM, Lohse M, Usadel B. Trimmomatic: a flexible trimmer for Illumina sequence data. Bioinformatics. 2014;30(15):2114–20. doi: 10.1093/bioinformatics/btu170 24695404 PMC4103590

[28] Vasimuddin Md, Misra S, Li H, Aluru S. Efficient Architecture-Aware Acceleration of BWA-MEM for Multicore Systems. In: 2019 IEEE International Parallel and Distributed Processing Symposium (IPDPS). IEEE. 2019. doi: 10.1109/ipdps.2019.00041

[29] Bankevich A, Nurk S, Antipov D, Gurevich AA, Dvorkin M, Kulikov AS, et al. SPAdes: a new genome assembly algorithm and its applications to single-cell sequencing. J Comput Biol. 2012;19(5):455–77. doi: 10.1089/cmb.2012.0021 22506599 PMC3342519

[30] Mikheenko A, Saveliev V, Gurevich A. MetaQUAST: evaluation of metagenome assemblies. Bioinformatics. 2016;32(7):1088–90. doi: 10.1093/bioinformatics/btv697 26614127

[31] Buchfink B, Xie C, Huson DH. Fast and sensitive protein alignment using DIAMOND. Nat Methods. 2015;12(1):59–60. doi: 10.1038/nmeth.3176 25402007

[32] Beier S, Tappu R, Huson DH. Functional Analysis in Metagenomics Using MEGAN 6. In: Charles TC, Liles MR, Sessitsch A, editors. Functional Metagenomics: Tools and Applications. Cham: Springer International Publishing; 2017. p. 65–74.

[33] Ondov BD, Bergman NH, Phillippy AM. Krona: Interactive Metagenomic Visualization in a Web Browser. In: Nelson KE, editor. Encyclopedia of Metagenomics. New York (NY): Springer New York; 2013. p. 1–8.

[34] Li H, Handsaker B, Wysoker A, Fennell T, Ruan J, Homer N, et al. The Sequence Alignment/Map format and SAMtools. Bioinformatics. 2009;25(16):2078–9. doi: 10.1093/bioinformatics/btp352 19505943 PMC2723002

[35] Hall RJ, Wang J, Todd AK, Bissielo AB, Yen S, Strydom H, et al. Evaluation of rapid and simple techniques for the enrichment of viruses prior to metagenomic virus discovery. J Virol Methods. 2014;195:194–204. doi: 10.1016/j.jviromet.2013.08.035 24036074 PMC7113663

[36] Conceição-Neto N, Zeller M, Lefrère H, De Bruyn P, Beller L, Deboutte W, et al. Modular approach to customise sample preparation procedures for viral metagenomics: a reproducible protocol for virome analysis. Sci Rep. 2015;5:16532. doi: 10.1038/srep16532 26559140 PMC4642273

[37] Beerenwinkel N, Günthard HF, Roth V, Metzner KJ. Challenges and opportunities in estimating viral genetic diversity from next-generation sequencing data. Front Microbiol. 2012;3:329. doi: 10.3389/fmicb.2012.00329 22973268 PMC3438994

[38] Gallego Romero I, Pai AA, Tung J, Gilad Y. RNA-seq: impact of RNA degradation on transcript quantification. BMC Biol. 2014;12:42. doi: 10.1186/1741-7007-12-42 24885439 PMC4071332

[39] Shiwa Y, Baba T, Sierra MA, Kim J, Mason CE, Suzuki H. Evaluation of rRNA depletion methods for capturing the RNA virome from environmental surfaces. BMC Res Notes. 2023;16(1):142. doi: 10.1186/s13104-023-06417-9 37420286 PMC10326927

