# Supplementary material for: Evaluation of metatranscriptomic sequencing protocols to obtain full-length RNA virus genomes from mammalian tissues
Source: PLoS One. 2025 May 30;20(5):e0324537. doi: 10.1371/journal.pone.0324537 (PMC12124746; doi:10.1371/journal.pone.0324537)

Supplementary materials

Table S1 Results of RNA purification and sequencing.

sample	volume (µL)	concentration (ng/µL)	quantity (ng)	RIN/RQN	extraction date	RNA pre-treatment assay	library preparation protocol	conc/tion post libr prep (ng/µL)	quantity post libr prep (ng)	mean fragm size (bp)	PF Clusters	% >= Q30 bases
**CRT125**	12	0.35	4.2	1	18/04/2016	method A	SPIA and Ovation UltraLow library construction	10	300	415	7675533	60.53
MOZ002	12	0.62	7.44	2	18/04/2016	method A						
MOZ091	12	0.41	4.92	1	18/04/2016	method A						
**MOZ135**	12	0.5	6	1	18/04/2016	method A	SPIA and Ovation UltraLow library construction	2.37	71.1	404	25652787	82.21
TA106	12	0.46	5.52	1	18/04/2016	method A						
TA531	12	0.67	8.04	1	18/04/2016	method A						
	**mean**	0.50	6.02	1.2				6.19	185.55	410	33328320	71.37
	**median**	0.48	5.76	1.0								
**CRT125**	30	118	3540	8.7	23/05/2017	method B	NEXTflexTM Rapid Illumina Directional RNA-Seq Library Prep Kit, Bioo Scientific	1.46	36.5	470	38253680	92.54
MOZ002	40	159	6360	5.5	11/05/2017	method B						
MOZ091	40	232	9280	4.4	08/05/2017	method B						
**MOZ135**	12	17	204	3.8	11/05/2017	method B	NEXTflexTM Rapid Illumina Directional RNA-Seq Library Prep Kit, Bioo Scientific	9	225	474	27237820	89.25
TA106	20	276	5520	2.3	18/05/2017	method B						
TA531	20	590	11800	8.8	23/05/2017	method B						
	**mean**	232	6117	5.6				5.23	130.75	472	65491500	90.90
	**median**	195.5	5940	4.95								

Table S2 Results of the comparison between SPAdes and metaSPAdes algorithms for the *de novo* assembly of the reads from the normalized subsets.
All statistics are based on contigs of size ≥ 100 bp, unless otherwise noted (e.g., "# contigs (≥ 0 bp)" include all contigs).

	Method A				Method B			
	CRT125		MOZ135		CRT125		MOZ135	
**Assembly**	SPAdes	metaSPAdes	SPAdes	metaSPAdes	SPAdes	metaSPAdes	SPAdes	metaSPAdes
**# contigs (>= 0 bp)**	891	1105	837	518	2977	3058	14466	14810
**# contigs (>= 1000 bp)**	8	5	16	12	47	44	78	54
**# contigs (>= 5000 bp)**	0	0	0	0	5	5	2	1
**Total length (>= 0 bp)**	233862	286703	175029	141036	972347	977612	4449547	4463960
**Total length (>= 1000 bp)**	12462	6361	26755	27617	111641	100564	134839	80570
**Total length (>= 5000 bp)**	0	0	0	0	41911	37389	11076	5482
**# contigs**	814	995	582	435	2937	2991	14302	14575
**Largest contig**	2102	1484	3024	4870	10864	9750	5635	5482
**Total length**	228583	278503	157652	135259	968966	972029	4435490	4444768
**N50**	262	270	279	282	286	283	290	286
**N90**	210	208	127	203	228	227	231	230
**auN**	384.9	348.8	566.4	818.5	832.9	728.3	393.6	361.6
**L50**	290	344	146	108	927	962	5308	5500
**L90**	683	821	436	339	2477	2522	12237	12472

Figure S1 Composition of ribovirus diversity and abundance in normalized datasets. Application of method A to sample CRT125 (A) and of method B to sample CRT125 (B), and of method A to sample MOZ135 (C), of method B to sample MOZ135 (D) are presented in Krona plots respectively with detailed classification within each riboviral families. The riboviral taxonomy is shown in nested sectors arranged from the top level of the hierarchy at the center (Root) and progressing outwards. The normalized count of contigs associated with each taxonomy is shown in brackets, while its corresponding percentage within the total contigs is presented alongside.

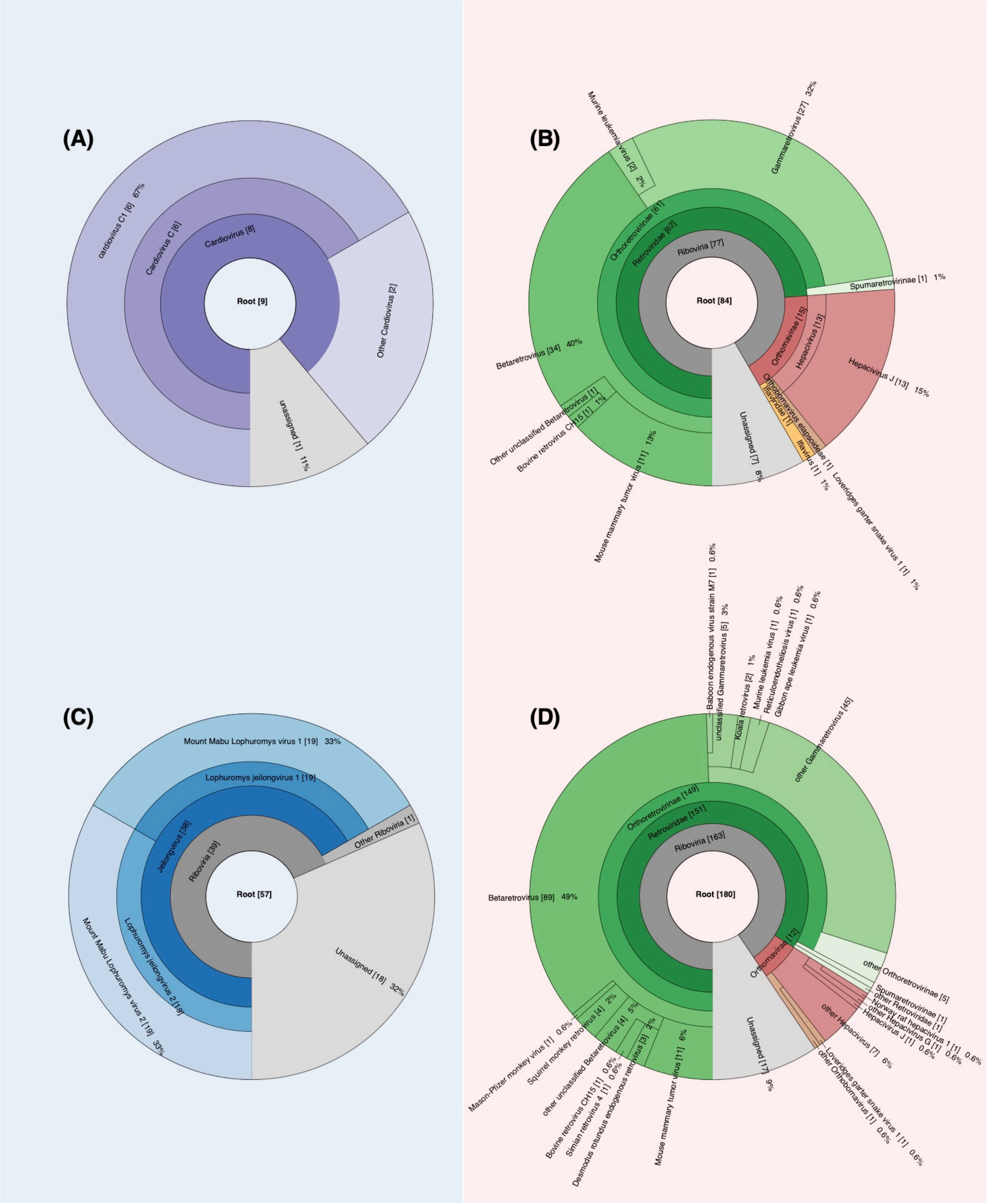

Supplement: S1 File — Supplementary materials: Table S1: Results of RNA purification and sequencing. Table S2: Results of the comparison between SPAdes and metaSPAdes algorithms for the de novo assembly of the reads from the normalized subsets. Figure S1: Composition of ribovirus diversity and abundance in normalized datasets. (PDF) [file pone.0324537.s001.pdf]
