# Supplementary material for: Evaluation of metatranscriptomic sequencing protocols to obtain full-length RNA virus genomes from mammalian tissues
Source: PLoS One. 2025 May 30;20(5):e0324537. doi: 10.1371/journal.pone.0324537 (PMC12124746; doi:10.1371/journal.pone.0324537)
Supplement: S2 File — Also available on protocols.io: https://dx.doi.org/10.17504/protocols.io.8ufhwtn (PDF) [file pone.0324537.s002.pdf]

Jan 24, 2020

High-quality RNA purification with on-column DNase treatment from tissue specimens

In 1 collection

DOI
dx.doi.org/10.17504/protocols.io.8ufhwtn

Magda Bletsa[1], Antonios Fikatas[2], Sophie Gryseels[2,3], Jan Felix Drexler[4], Philippe Lemey[2], Yiqiao Li[2]

[1]National and Kapodistrian University of Athens;
[2]Department of Microbiology, Immunology and Transplantation, Rega Institute, KU Leuven – University of Leuven, Leuven, Belgium;
[3]Department of Ecology and Evolutionary Biology, University of Arizona, Tucson, AZ, USA;
[4]Charité-Universitätsmedizin Berlin, corporate member of Freie Universität Berlin Humbolt-Universität zu Berlin and Berlin Institute of Health, Institute of Virology, Berlin, Germany

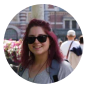

Magda Bletsa
Hellenic Pasteur Institute, National and Kapodistrian Univer...

OPEN ACCESS

DOI: dx.doi.org/10.17504/protocols.io.8ufhwtn

Protocol Citation: Magda Bletsa, Antonios Fikatas, Sophie Gryseels, Jan Felix Drexler, Philippe Lemey, Yiqiao Li 2020. High-quality RNA purification with on-column DNase treatment from tissue specimens . **protocols.io**
https://dx.doi.org/10.17504/protocols.io.8ufhwtn

License: This is an open access protocol distributed under the terms of the **Creative Commons Attribution License**, which permits unrestricted use, distribution, and reproduction in any medium, provided the original author and source are credited

Protocol status: Working

This protocol can be used for total RNA purification from tissue samples. It includes an intermediate on-column DNase treatment, which results in higher RNA yield and purity. This method is mainly optimised to obtain hepacivirus RNA extracts for whole-genome sequencing, but it can also be used for any viral RNA purification from samples with lower viral loads.

Created: October 29, 2019

Last Modified: January 24, 2020

Protocol Integer ID: 29287

Keywords: RNA extraction, hepacivirus, tissue

Abstract

This protocol can be used for total RNA purification from tissue samples. It includes an intermediate on-column DNase treatment, which results in higher RNA yield and purity. This method is mainly optimised to obtain hepacivirus RNA extracts for whole-genome sequencing, but it can also be used for any viral RNA purification from samples with lower viral loads.

Guidelines

- During tissue handling, all procedures should be carried out as quickly as possible.
- Only RNA molecules > 200 nucleotides are purified.
- Do not overload the RNeasy spin column (maximum capacity of 700 µL).
- Buffer RLT may form a precipitate upon storage. Re-dissolve by putting the bottle under warm water for a few minutes.
- Perform all steps at Room temperature , unless otherwise stated.
- Always use new collection tubes to eliminate any possible contamination.

Materials

MATERIALS

- RNeasy Mini Kit **Qiagen Catalog #**74104
- RNase-Free DNase Set **Qiagen Catalog #**79254
- Precellys CK28 Lysing Kit Hard Tissue Homogenizing Bertin **VWR International Catalog #**10144-516

STEP MATERIALS

- Precellys CK28 Lysing Kit Hard Tissue Homogenizing Bertin **VWR International Catalog #**10144-516

Protocol materials

- RNeasy Mini Kit **Qiagen Catalog #**74104 Materials
- RNase-Free DNase Set **Qiagen Catalog #**79254 Materials
- Precellys CK28 Lysing Kit Hard Tissue Homogenizing Bertin **VWR International Catalog #**10144-516

In Materials, Materials, Step 1

Before start

- When using Buffer RPE for the first time, add 4 volumes of ethanol (100%).
- Prepare DNase I stock solution of RNase-free DNase set as described:
  Do not open the glass vial. Transfer 550 µL of the RNase-free water provided into a 1.5 mL Eppendorf tube.
  Using a needle and a syringe inject the water into the lyophilised DNase I glass vial. Mix gently by inverting the vial and make sure the powder on the sides of the vial is all well dissolved. **DO NOT VORTEX**.
  For long-term storage of DNase I, remove the stock solution from the glass vial, using the syringe. Divide it into 100 µL aliquots and store at -- 20 °C °C for up to 9 months. Thawed aliquots can be stored at 4 °C for up to 6 weeks. Do not refreeze the aliquots after thawing.

Sample homogenisation

1 Add 600 µL RLT Buffer to the Precellys lysate tubes.

Precellys CK28 Lysing Kit Hard Tissue Homogenizing Bertin **VWR International Catalog #**10144-516

2 Excise a lentil-sized piece of tissue (maximum amount of 20 mg for RNAlater stabilized tissues and 30 mg for fresh or frozen tissues) and transfer it quickly to the lysate tubes. Make sure that all tissues are immersed into the RLT reagent.

3 Place tubes on dry ice for 00:02:00 (or until frozen) and then thaw quickly.

4 Immediately after thawing disrupt the tissues using a conventional rotor-stator homogeniser (Minilys). The recommended lysis should be performed at medium speed (4000 rpm) for 00:02:00 .

Equipment	
**Minilys Personal Homogeniser**	NAME
Tissue homogeniser	TYPE
Bertin Instruments	BRAND
P000673-MLYS0-A	SKU
https://www.bertin-instruments.com/product/sample-preparation-homogenizers/minilys-tissue-homogenizer/	LINK

5 Place tubes on dry ice for 00:02:00 (or until frozen) and then thaw quickly. Check the results and repeat the homogenisation until no more visible fragments are present.

6 Centrifuge the lysate for 00:03:00 at full speed. Carefully remove the supernatant by pipetting, and transfer it to a new 1.5 mL Eppendorf tube.

RNA binding

7 Add 600 µL 70% ethanol to the supernatant, and mix immediately by pipetting 5 times. **DO NOT VORTEX OR CENTRIFUGE**. Proceed immediately to the next step.

8 Transfer 700 µL of the sample to an RNeasy spin column placed in a 2 mL collection tube. Centrifuge for 00:00:30 at 10000 rpm
If the sample is more than 700 µL , change collection tubes and transfer the rest to the spin column and centrifuge again.
Discard the collection tubes and replace with new ones.

9 Add 350 µL Buffer RW1 to the RNeasy spin column and centrifuge for 00:00:30 at 10000 rpm to wash the spin column membrane. Change collection tubes.

DNase treatment

10 Prepare the DNase treatment master mix, as described:
For each sample, add 10 µL DNase I stock solution to 70 µL Buffer RDD. Mix by gently inverting the tube, and spin down briefly. **DO NOT VORTEX**.

11 Add the 80 µL DNase I incubation mix directly to the RNeasy spin column membrane, and place on benchtop at Room temperature for 00:15:00 .

Washing steps

12 Add 350 µL Buffer RW1 to the RNeasy spin column and centrifuge for 00:00:30 at 10000 rpm to wash the spin column membrane. Change collection tubes.

13 Add 500 µL Buffer RPE to the RNeasy spin column and centrifuge for 00:00:30 at 10000 rpm . Change collection tubes.

14 Add 500 µL Buffer RPE to the RNeasy spin column and centrifuge for 00:02:00 at 10000 rpm . Change collection tubes.

Dry centrifugation

15 Place the RNeasy spin column in a new collection tube and centrifuge at full speed for 00:05:00 If there is still much liquid passing through the column, change collection tubes and centrifuge for 00:01:00 .

Elution and storage

16 Place the spin column in a new 1.5 mL Eppendorf tube. Add 50 µL RNase-free water directly to the spin column membrane. Incubate for 00:05:00 at Room temperature . Then centrifuge for 00:01:00 at 10000 rpm to elute the RNA.

17 Repeat step 16. Use a new 1.5 mL tube for another elution round of 50 µL .

18 Store at -- 20 °C . Optionally, you can put the column back to the corresponding collection tube and store them at -- 20 °C for another future elution. Even after the 3rd or 4th elution time, there are still some RNA molecules passing through the column.
