# Supplementary material for: Evaluation of metatranscriptomic sequencing protocols to obtain full-length RNA virus genomes from mammalian tissues
Source: PLoS One. 2025 May 30;20(5):e0324537. doi: 10.1371/journal.pone.0324537 (PMC12124746; doi:10.1371/journal.pone.0324537)
Supplement: S3 File — Also available on protocols.io: https://dx.doi.org/10.17504/protocols.io.14egn6w96l5d/v1 (PDF) [file pone.0324537.s003.pdf]

Sep 20, 2024

NGS library preparation using Ovation RNA-Seq System V2 (M01206 v9) and Ovation Ultralow System V2 (M01437 v2) for animal tissue samples

Forked from a private protocol

DOI
dx.doi.org/10.17504/protocols.io.14egn6w96l5d/v1

Ine Boonen[1], Magda Bletsa[2,3], Yiqiao Li[4], Philippe Lemey[1]

[1]Department of Microbiology, Immunology and Transplantation, Rega Institute, KU Leuven – University of Leuven, Leuven, Belgium;
[2]Bioinformatics and Applied Genomics Unit, Department of Microbiology, Hellenic Pasteur Institute;
[3]Department of Hygiene, Epidemiology and Medical Statistics, National and Kapodistrian University of Athens;
[4]Ruijin Hospital, Shanghai Jiao Tong University School of Medicine

Ine Boonen
KU Leuven

OPEN ACCESS

DOI: dx.doi.org/10.17504/protocols.io.14egn6w96l5d/v1

Protocol Citation: Ine Boonen, Magda Bletsa, Yiqiao Li, Philippe Lemey 2024. NGS library preparation using Ovation RNA-Seq System V2 (M01206 v9) and Ovation Ultralow System V2 (M01437 v2) for animal tissue samples. **protocols.io**
https://dx.doi.org/10.17504/protocols.io.14egn6w96l5d/v1

License: This is an open access protocol distributed under the terms of the **Creative Commons Attribution License**, which permits unrestricted use, distribution, and reproduction in any medium, provided the original author and source are credited

Protocol status: Working
We use this protocol and it's working

Created: July 02, 2024

Last Modified: September 20, 2024

Protocol Integer ID: 102727

Keywords: NGS, Library Preparation, Tissue samples, RNA Sequencing, Virus, Metagenomic

Abstract

This protocol is used for successful NGS library preparation from total RNA of animal tissue samples.
With this method we obtained full-length paramyxovirus genomes using the Illumina sequencing platform. It works most efficiently on RNA viruses possessing a polyA tail, but it can also be used for sequencing any other RNA viral sequences originating from tissue samples.

Guidelines

- Thaw components used in each step and immediately place them on ice. Always keep thawed reagents and reaction tubes on ice unless otherwise instructed. Do not thaw all reagents at once.
- After thawing and mixing buffer mixes, if any precipitate is observed, re-dissolve the precipitate completely prior to use. You may gently warm the buffer mix for 2 minutes at room temperature followed by brief vortexing.
- Keep enzyme mixes on ice after briefly spinning to collect the contents. Do not vortex enzyme mixes nor warm any enzyme or primer mixes.
- When instructed to mix via pipetting, gently aspirate and dispense a volume that is at least half of the total volume of the reaction mix.
- Use only fresh ethanol stocks to prepare the dilutions of ethanol washes throughout the purification protocols.
- Return all reagents to their appropriate storage conditions promptly after use unless otherwise instructed.
- Total RNA input must be between 500 pg and 100 ng.
- RNA samples must be free of contaminating proteins and other cellular material, organic solvents (including phenol and ethanol) and salts used in many RNA isolation methods. If a method such as Trizol is used, we recommend using a column purification after isolation.

Materials

Ovation RNA-Seq System V2 (M01206 v9)

MinElute Reaction Cleanup Kit **Qiagen Catalog #**28204

Ovation® Ultralow V2 DNA-Seq Library Preparation Kit **Tecan Catalog #** 0344NB-A01

Protocol materials

MinElute Reaction Cleanup Kit **Qiagen Catalog #**28204 Step 44

Ovation® Ultralow V2 DNA-Seq Library Preparation Kit **Tecan Catalog #** 0344NB-A01 Step 65

Safety warnings

Due to the high sensitivity inherent in this amplification system, we strongly recommend taking measures to minimize the potential for the carryover of previously amplified SPIA cDNA into new amplification reactions. The two steps to accomplish this are:

1. Designating separate workspaces for “pre-amplification” and “post-amplification” steps and materials.
2. Implementing routine clean-up protocols for workspaces as standard operating procedures.

Before start

- Check RNA sample quality prior to library preparation. RNA sample quality may vary between sample preparations.
- Ensure pipettes are properly calibrated as library preparations are highly sensitive to pipetting error.

A. Sample Preparation for Ovation RNA-Seq system V2

1 Remove Nuclease-free water (Green: D1) from the freezer and let it thaw at Room temperature .

OVATION RNA-SEQ SYSTEM V2

2 The following kit was used to prepare cDNA from RNA prior to the library preparation step: Ovation RNA-SEQ System V2 (M01206 v9)

A. Sample Preparation for Ovation RNA-Seq system V2

3 Aliquot each total RNA input sample (500 pg-100 ng) into a 0.2 ml strip tube or a 96-well plate. Input volume can not exceed 5 µL .

4 Dilute the RNA with D1 to a final volume of 5 µL .

5 Note: Keep D1 at Room temperature for use in next sections

B. First Strand cDNA Synthesis

6 Remove the First Strand Primer Mix (blue: A1) and First Strand Buffer mix (Blue: A2) from the freezer. Let it thaw at Room temperature . Mix by vortexing, spin down and place on ice.

7 Remove First Strand Enzyme Mix (blue: A3) from the freezer, spin down and place on ice.

8 Add 2 µL of A1 to 5 µL of each input RNA for a total of 7 µL . Mix by pipetting 5 times, spin down and place on ice.

9 Place the tubes in a preheated thermal cycler programmed to run the Primer annealing program:

Primer annealing

For RNA inputs ≤ 1 ng

Step 1	65°C	2 min
Step 2	4°C	Hold

For RNA inputs ≥ 1 ng

Step 1	65°C	5 min
Step 2	4°C	Hold

10 Remove the tubes from the thermal cycler and place on ice.

11 Once the Primer annealing (step 8) is complete, prepare a master mix of A2 and A3 as follows:

First Strand Master Mix

Reagent	1x Reaction Volume
A2: First Strand Buffer Mix	2.5 µL
A3: First Strand Enzyme Mix	0.5 µL
**Total Volume**	**3 µL**

Mix well by pipetting 5 times, spin down and place on ice.

12 Add 3 µL of First Strand Master Mix to each sample tube for a total of 10 µL. Mix by pipetting 5 times, spin and place on ice.

13 Place the tubes in a preheated thermal cycler programmed to run the First Strand synthesis program:

First Strand Synthesis

Step 1	4°C	1 min
Step 2	25°C	10 min
Step 3	42°C	10 min
Step 4	70°C	15 min
Step 5	4°C	Hold

14 Remove the tubes from the thermal cycler, spin to collect all droplets from the wall of the tubes and place them on ice.

C. Second strand cDNA Synthesis

15 Remove Agencourt beads from the fridge and place on the bench top to reach Room temperature for use in the next step. (At least 00:30:00 before section D) 30m

16 Remove the Second Strand Buffer Mix (Yellow: B1) from the freezer. Let it thaw at Room temperature . Mix by vortexing, spin down and place on ice.

17 Remove Second Strand Enzyme Mix (Yellow: B2) from the freezer, spin down and place on ice.

18 Prepare a master mix of B1 and B2 as follows:

Second Strand Master Mix

Reagent	1x Reaction Volume
B1: Second Strand buffer Mix	9.7 µL
B2: Second Strand Enzyme Mix	0.3 µL
**Total Volume**	**10 µL**

Mix well by pipetting 5 times, spin down and place on ice.

19 Add 10 µL of Second Strand Master Mix to each sample tube for a total of 20 µL
Mix well by pipetting 5 times, spin down and place on ice.

20 Place the tubes in a preheated thermal cycler programmed to run the Second Strand synthesis program:

Second Strand Synthesis

Step 1	4°C	1 min
Step 2	25°C	10 min
Step 3	50°C	30 min
Step 4	80°C	20 min
Step 5	4°C	Hold

21 Remove the tubes from the thermal cycler, spin to collect all droplets from the wall of the tubes and place them in a rack on the bench top.

D. cDNA Purification

22 Ensure the Agencourt beads have completely reached Room temperature before proceeding.

23 Prepare a 70% Ethanol wash solution
NOTE:It is critical this solution to be prepared fresh on the same day of the experiment from a recently opened stock container.
Measure both water and ethanol components carefully prior to mixing. Failure to do so can result in a higher than anticipated aqueous content, which may reduce amplification yield.

24 Resuspend the beads by vortexing. Ensure that the beads are fully resuspended before adding them to the sample. After resuspending DO NOT spin the beads

25 At Room temperature , add 32 µL (1.6 volumes) of Agencourt beads to each reaction and mix by gently pipetting up and down 10 times.

26 Incubate at Room temperature for 00:10:00 10m

27 Transfer tubes to the magnet and let them stand for 00:05:00 until the solution of the beads seems completely clear. 5m

28 Keeping the tubes on the magnet, add 200 µL of freshly prepared 70% ethanol and allow to stand for 00:00:30 . 30s
NOTE: The beads should not disperse; instead, they will stay on the walls of the tubes. Significant loss of beads at this stage will impact cDNA yields, so ensure beads are not removed with the binding buffer or the washes.

29 Remove the 70% ethanol wash using a pipette.

30 Repeat the previous washing steps two more times.

31 With the final wash, it is critical to remove as much of the ethanol as possible. Use at least 2 pipetting steps and allow excess ethanol to collect at the bottom of the tubes after removing most of the ethanol in the first pipetting step. Try to remove last bits of ethanol with a smaller pipette.

32 Air-dry the bead on the magnet for 15 - 20 min. Inspect each tube carefully to ensure that all the ethanol has evaporated.
IMPORTANT!: It is critical that all residual ethanol be removed prior to continuing with SPIA amplification.

33 Continue immediately to the SPIA Amplification protocol with the cDNA still bound to the dry beads.

E. SPIA Amplification

34 Remove the SPIA Primer Mix (Red: C1) and the SPIA Buffer Mix (Red: C2) from the freezer. Let it thaw at Room temperature . Mix by vortexing, spin down and place on ice.

35 Remove SPIA Enzymer Mix (Red: C3) from the freezer, mix the contents by inverting gently 5 times without introducing bubbles. Spin down and place on ice.

36 Prepare the SPIA master mix by sequentially combining C2, C1 and C3 as follows:

SPIA Master Mix

Reagent	1x Reaction Volume
C2: SPIA Buffer Mix	20 µL
C1: SPIA Primer Mix	10 µL
C3: SPIA Enzyme Mix	10 µl
**Total Volume**	**40 µL**

NOTE: Make sure to add C3 last.

37 Add 40 µL of the SPIA Master Mix to each tube containing the double-stranded cDNA bound to the dried beads. Use a pipette set to 30 µL and mix thoroughly by pipetting at least 8-10 times. Attempt to get the majority of the beads in suspension and remove most of the beads from the tube walls.
NOTE: The beads may not form a perfectly uniform suspension, but this will not affect the reaction. The addition of SPIA master mix will elute the cDNA from the beads.

38 Place the tubes in a pre-warmed thermal cycler programmed to run the SPIA amplification program:

SPIA amplification

Step 1	4°C	1 min
Step 2	47°C	60 min
Step 3	80°C	20 min
Step 4	4°C	Hold

39 Remove the tubes from thermal cycler, spin to collect condensation and place on ice.

40 **IMPORTANT!**: At this point the tubes should be removed from the pre-amplification workspace. Carry out all remaining steps in a post-amplification workspace using dedicated post-amplification consumables and equipment. Take care to avoid the introduction of previously amplified cDNA into your pre-amplification workspace.

41 Transfer tubes to the magnet and let them stand for 00:05:00 to completely clear the solution of beads. 5m

42 Carefully transfer 40 µL of the cleared supernatant containg the SPIA cDNA to a fresh tube.

43 Continue immediatly with the Purification of SPIA cDNA protocol or store the reaction products at -20 °C .

F. Purification of SPIA cDNA

44 For purification the MinElute Reaction Cleanup Kit **Qiagen Catalog #**28204 was used following the manufacturer's guidelines. This kit was chosen due to its low elution.
IMPORTANT!:
- Buffer ERC is considered hazardous according to QIAGEN, and an SDS should be consulted.
- Add the appropriate amount of 100% ethanol to Buffer PE before use (see bottle label for volume). Failure to do so will result in low amplification yields.
- All centrifugation steps are carried out at maximum speed in a conventional tabletop microcentrifuge at Room temperature .

45 Add 300 µL of Buffer ERC from the QIAGEN kit into a clean, labeled 1.5 ml microcentrifuge tube.

46 Transfer the entire volume (40 µL) of the SPIA reaction to the 1.5 ml tube containing buffer ERC.

47 Vortex for 5s and then spin briefly.

48 Label a MinElute spin column and place it into a collection tube.

49 Load the sample/buffer mixture onto the column and centrifuge 00:01:00 at maximum speed. 1m

50 Place the column in a new collection tube and discard the old collection tube with the flow-through.

51 Add 750 µl of Buffer PE to the column and centrifuge for 00:01:00 at maximum speed. 1m

52 Place the column in a new collection tube and discard the old collection tube with the flow-through.

53 Centrifuge the column for an additional 00:02:00 at maximum speed to remove all residual buffer PE. 2m

54 Discard flow-through along with collection tube. Blot the column tip onto clean absorbent paper to remove any residual wash buffer from the tip of the column.

55 Place the column into a clean labeled 1.5 ml microcentrifuge tube. Add 22 µL of Room temperature 1x TE or Buffer EB to the center of each column.

56 Let the column stand for 00:01:00 at Room temperature . 1m

57 Centrifuge for 00:01:00 at maximum speed. 1m

58 Measure the volume recovered. There should be approximately 20 µl of purified SPIA cDNA. Then discard the column.

59 Continue with the SPIA cDNA yield and Purity Protocol or store the purified SPIA cDNA at -20 °C

G. SPIA cDNA Yield and Purity

60 Mix the purified SPIA cDNA sample by brief vortexing. Spin down contents.

61 Measure the absorbance at 230, 260, 280 and 320 nm on a spectophotometer. You may need to make a 1:20 dilution of the cDNA in water prior to measuring the absorbance.

62 **Purity:**
Subtract the A320 value from the A230, A260 and A280 values. The adjusted (A260–A320) / (A280–A320) ratio should be greater than 1.8. The adjusted (A260–A320)/(A230–A320) ratio should be greater than 2.

63 **Yield:**
Assume 1 A260 unit = 50 µg/mL.
To calculate: (A260–A320 of diluted sample) X (dilution factor) X 50 (concentration in µg/mL of a 1 A260 unit solution) X 0.02 (final volume in mL) = total yield in micrograms
NOTE: If there is not available a spectrophotometer, you can use any other type of quantitation system to quantify the SPIA amplification products (i.e. Qubit, QuantiFluor).

64 The purified cDNA may be stored at -20 °C

OVATION ULTRALOW SYSTEM V2

65 The following kit was used to make libraries for sequencing from the previously prepared cDNA.
Ovation® Ultralow V2 DNA-Seq Library Preparation Kit **Tecan** **Catalog #** 0344NB-A01
IMPORTANT!: Make sure to use reagents from the right kit. The reagents in the ovation RNA-seq system V2 product can be similar to reagents in this kit, however, unless the component part numbers are identical, these reagents do not have exactly the same composition and therefore, are not interchangeable. For example A1 from one kit with A1 from another kit.
NOTE: Remove Agencourt beads from fridge and Nuclease-free Water (green: D1) from freezer and place them on bench top.

H. DNA Fragmentation

66 No fragmentation step was performed, since the input cDNA fragment sizes were relatively short. Therefore, we also skipped the DNA purification step, which is normally performed after the DNA fragmentation, and directly proceeded to the end repair step.

I. End Repair

67 Remove the End Repair Buffer Mix (Blue:ER1). Let it thaw at Room temperature . Mix by vortexing, spin down and place on ice.

68 Remove End Repair Enzyme Mix (Blue: ER2) and End Repair Enhancer (blue: ER3) from the freezer. Spin down and place on ice.

69 Obtain 10 µL of the cDNA sample (10 pg – 100 ng) that was previously stored at -20 °C . Alternatively, place 10 pg – 100 ng of DNA in 10 µL of low-EDTA TE buffer or Nuclease-free Water in a PCR tube.

70 Prepare the End repair master mix by combining ER1, ER2 and ER3 as follows:

End Repair Master Mix

Reagent	1x Reaction Volume
ER1: Repair Buffer Mix	3.5 µL
ER2: End Repair Enzyme Mix	0.5 µL
ER3: End Repair Enhancer	1 µl
**Total Volume**	**5 µL**

71 Add 5 µL of the End Repair Master Mix to each sample tube and mix by pipetting. Spin the tubes down and place on ice.

72 Place the tubes in a preheated thermal cycler programmed to run the End Repair amplification program:

End Repair amplification

Step 1	25°C	30 min
Step 2	70°C	10 min
Step 3	4°C	Hold

73 Remove the tubes from the thermal cycler, spin to collect all droplets from the wall of the tubes and place them on ice. Continue immediatly with the Ligation Protocol.

J. Ligation

74 Remove the Ligation Buffer Mix (Yellow: L1) and Ligation Adaptor Mix (Yellow:L2) from the freezer. Let it thaw On ice . Mix by vortexing, spin down and place on ice.

75 Remove Ligation Enzyme Mix (Yellow:L3) from the freezer. Spin down and place on ice.

76 Add L2 to each sample as follows:

- If using adaptors from tubes (0344NB-08), add 6 µL of the appropriate L2 Ligation Adaptor Mix to each sample. Mix thoroughly by pipetting.
- If using an adaptor plate (0344-32, 0344NB-32 or 0344NB-A01), add the entire 15 µL of sample to the appropriate adaptor well, mix well by pipetting, then transfer the entire sample to a PCR tube.

NOTE: All samples on a sequencing run should have unique ligation adaptors.

77 Prepare the Ligation master mix by combining D1, L1 and L3 as follows:

Ligation Master Mix

Reagent	1x Reaction Volume
D1: Water	1.5 µL
L1: Ligation Buffer Mix	6 µL
L3: Ligation Enzyme Mix	1.5 µl
**Total Volume**	**9 µL**

NOTE: L1 is very viscous. Please be sure to pipette this reagent slowly.

78 Add 9 µL of the Ligation Master Mix to each sample tube and mix by pipetting. Spin the tubes down and place on ice. Proceed immediatly with the incubation.

79 Place the tubes in a preheated thermal cycler programmed to run the End Repair amplification program:

Ligation amplification

Step 1	25°C	30 min
Step 2	70°C	10 min
Step 3	4°C	Hold

80 Remove the tubes from the thermal cycler, spin to collect all droplets from the wall of the tubes and place on ice. Continue immediately with the Ligation Purification Protocol.

K. Ligation Purification

81 Prepare a 70% ethanol wash solution. It is critical this solution to be prepared fresh on the same day of the experiment from a recently opened stock container. Measure both the ethanol and the water components carefully prior to mixing. Failure to do so can result in a higher than anticipated aqueous content, which may reduce yield.

1.5 mL wash solution per sample is enough for all the bead purification steps.

82 Ensure the Agencourt beads and Nuclease-free Water (D1) have completely reached Room temperature before proceeding. Make sure beads are resuspended by inverting and tapping the tube. After resuspending, do not spin the beads.

83 Add 70 µL of Room temperature D1 to each ligation reaction.

84 At room temperature, add 80 µL (0.8 volumes) of the bead suspension to each tube and mix by pipetting 10 times.

85 Incubate at Room temperature for 00:10:00 . 10m

86 Transfer the PCR tubes containing the bead-sample mixture to the magnet and let them stand for 00:05:00 to completely clear the solution of beads.

87 Carefully remove 160 µL of the binding buffer and discard it. Leaving some of the volume behind minimizes bead loss at this step.

88 With the tubes still on the magnet, add 200 µL of freshly prepared 70% ethanol and allow to stand for 00:00:30 .

89 Remove the 70% ethanol wash using a pipette.

90 Repeat the 70% ethanol wash one more time, for a total of two washes.

91 With the final wash, it is critical to remove as much of the ethanol as possible. Use at least 2 pipetting steps and allow excess ethanol to collect at the bottom of the tubes after removing most of the ethanol in the first pipetting step. Try to remove last bits of ethanol with a smaller pipette.

92 Air dry the beads on the magnet for 00:10:00 . Inspect each tube carefully to ensure that all the ethanol has evaporated. 10m

93 Add 40 µL Room temperature 1X low-EDTA TE buffer or Nuclease-free Water (green: D1) to the dried beads. Mix thoroughly to ensure all the beads are resuspended and let them stand on the bench top for 00:03:00 3m

94 Transfer the tubes to the magnet and let them stand for 00:03:00 to completely clear the solution of beads. 3m

95 Carefully remove 35 µL of the eluate, ensuring as few beads as possible are carried over, then transfer to a fresh set of PCR tubes and place on ice.

96 Set aside the Agencourt beads and 70% ethanol at room temperature for use in the Amplified Library Purification protocol. Also set aside the Nuclease-free water (green:D1) for use throughout the protocol.

97 Continue immediately to the Library Amplification Protocol.

L. Library Amplification

98 Remove the Amplification Buffer Mix (Red: P1), Amplification Primer Mix (Red: P2) from the freezer. Let it thaw at Room temperature . Mix by vortexing, spin down and place on ice.

99 Remove Amplification Enzyme Mix (Red: P3) from the freezer. Spin down and place on ice.

100 Prepare the Ligation master mix by sequentially combining P1 and P2. Add P3 last, as follows, and mix well, taking care to avoid bubbles. Then spin down and place on ice.

Amplification Master Mix

Reagent	1x Reaction Volume
P1: Amplification Buffer Mix	12.75 µL
P2: Amplification Primer Mix	1.25 µL
P3: Amplification Enzyme Mix	1 µl
**Total Volume**	**15 µL**

101 On ice Add 15 µL of the Amplification Master Mix to each sample tube and mix by pipetting. Spin the tubes down and place on ice.

102 Place the tubes in a preheated thermal cycler programmed to run the End Repair amplification program:

Library amplification

Step 1	72°C	2 min	
Step 2	95°C	3 min	
Step 3	98°C	20s	8-15 cycles
Step 4	65°C	30s	
Step 5	72°C	30s	
Step 6	72°C	1 min	
Step 7	4°C	Hold	

IMPORTANT!: The number of cycles used for PCR amplification depends on the starting amount of genomic DNA. Please refer to the table below for a general guide to choosing the appropriate number of cycles for the PCR amplification reaction. Alternatively, real-time PCR can be used to determine the appropriate number of PCR cycles.

Starting Input	PCR Cycles
< 1ng	May require optimization
1 - 10 ng	13-15
10 – 50 ng	10-12
50 – 100 ng	7-9

103 Remove the tubes from the thermal cycler, spin to collect all droplets from the wall of the tubes and place on ice. Continue immediately with the Amplified Library Purification Protocol.

M. Amplified Library Purification

2m

104 Take Agencourt beads and 70% ethanol that we used earlier. Ensure they are at Room temperature before proceeding.

105 Make sure beads are resuspended by inverting and tapping the tube. After resuspending, do not spin the beads.

106 At room temperature, add 50 µL (1 volume) of the bead suspension to each tube and mix by pipetting 10 times.

107 Incubate at Room temperature for 00:10:00 .

108 Transfer the PCR tubes containing the bead-sample mixture to the magnet and let them stand for 00:05:00 to completely clear the solution of beads.

109 Carefully remove 85 µL of the binding buffer and discard it. Leaving some of the volume behind minimizes bead loss at this step.

110 With the tubes still on the magnet, add 200 µL of freshly prepared 70% ethanol and allow to stand for 00:00:30 .

111 Remove the 70% ethanol wash using a pipette.

112 Repeat the 70% ethanol wash one more time, for a total of two washes.

113 With the final wash, it is critical to remove as much of the ethanol as possible. Use at least 2 pipetting steps and allow excess ethanol to collect at the bottom of the tubes after removing most of the ethanol in the first pipetting step. Try to remove last bits of ethanol with a smaller pipette.

114 Air dry the beads on the magnet for 00:10:00 . Inspect each tube carefully to ensure that all the ethanol has evaporated.

115 Add 33 µL Room temperature 1X low-EDTA TE buffer or Nuclease-free Water (green: D1) to the dried beads. Mix thoroughly to ensure all the beads are resuspended.

116 Transfer the tubes to the magnet and let them stand for 00:02:00 to completely clear the solution of beads. 2m

117 Carefully remove 30 µL of the eluate, ensuring as few beads as possible are carried over, then transfer to a fresh set of PCR tubes and place on ice.

118 Library can now be stored at -20 °C

Protocol references

Ovation RNA-Seq System V2, M01206v9, NuGEN, TECAN
Ovation Ultralow System V2, M01379v5.1, NuGEN, TECAN
