# Supplementary material for: Evaluation of metatranscriptomic sequencing protocols to obtain full-length RNA virus genomes from mammalian tissues
Source: PLoS One. 2025 May 30;20(5):e0324537. doi: 10.1371/journal.pone.0324537 (PMC12124746; doi:10.1371/journal.pone.0324537)
Supplement: S4 File — Also available on protocols.io: https://dx.doi.org/10.17504/protocols.io.q26g74843gwz/v1 (PDF) [file pone.0324537.s004.pdf]

Jul 18, 2022

NGS library preparation using NEXTFLEX Rapid Directional RNAseq kit (NOVA-5138-08) for animal tissue samples

DOI
dx.doi.org/10.17504/protocols.io.q26g74843gwz/v1

Yiqiao Li[1], Magda Bletsa[2], Ine Boonen[1], Philippe Lemey[1]

[1]Department of Microbiology, Immunology and Transplantation, Rega Institute, KU Leuven – University of Leuven, Leuven, Belgium;
[2]National and Kapodistrian University of Athens

KU Leuven

Yiqiao Li
Ruijin Hospital, Shanghai Jiao Tong University School of Med...

OPEN ACCESS

DOI: dx.doi.org/10.17504/protocols.io.q26g74843gwz/v1

Protocol Citation: Yiqiao Li, Magda Bletsa, Ine Boonen, Philippe Lemey 2022. NGS library preparation using NEXTFLEX Rapid Directional RNAseq kit (NOVA-5138-08) for animal tissue samples. **protocols.io**
https://dx.doi.org/10.17504/protocols.io.q26g74843gwz/v1

Manuscript citation:
NEXTFLEX Rapid Directional RNAseq kit (NOVA-5138-08)

License: This is an open access protocol distributed under the terms of the **Creative Commons Attribution License**, which permits unrestricted use, distribution, and reproduction in any medium, provided the original author and source are credited

Protocol status: Working
We use this protocol and it's working properly for NGS aiming to generate complete hepacivirus genome from animal samples.

Created: January 19, 2022

Last Modified: July 18, 2022

Protocol Integer ID: 57131

Keywords: NGS, library preparation, tissue samples, RNA sequencing, virus, metagenomic

Disclaimer

DISCLAIMER – FOR INFORMATIONAL PURPOSES ONLY; USE AT YOUR OWN RISK

The protocol content here is for informational purposes only and does not constitute legal, medical, clinical, or safety advice, or otherwise; content added to **protocols.io** is not peer reviewed and may not have undergone a formal approval of any kind. Information presented in this protocol should not substitute for independent professional judgment, advice, diagnosis, or treatment. Any action you take or refrain from taking using or relying upon the information presented here is strictly at your own risk. You agree that neither the Company nor any of the authors, contributors, administrators, or anyone else associated with **protocols.io**, can be held responsible for your use of the information contained in or linked to this protocol or any of our Sites/Apps and Services.

Abstract

This protocol is used for successful NGS library preparation from total RNA of animal tissue samples.
This method is mainly optimised to obtain full-length hepacivirus genomes using the Illumina NGS sequencing platform, but it also can be used for sequencing any other RNA virus originating from tissue samples.

Image Attribution

NEXTFLEX Rapid Directional RNAseq kit (NOVA-5138-08)

Guidelines

- rRNA-depleted RNA input: ~1ng - 100 ng (Please check the manufacturer's protocol for other type of RNA inputs). Adjust to 14µL by nuclease-free water. The procedure is ideal for insert sizes of >150 bp.
- This kit contains a single Barcoded Adapter. To enable multiplexing, please use the appropriate combination of NEXTflex RNA-Seq Barcodes during the Adapter Ligation step.
- Try to maintain a laboratory temperature of 20°–25°C (68°–77°F).

Materials

- NEXTflex Rapid Directional RNAseq kit, PerkinElmer (NOVA-5138-08, 48 rxns)
- NEXTflex RNA-Seq Barcodes – 6 / 12 / 24 / 48 (Cat # 512911, 512912, 512913, 512914)
- 100 % Ethanol
- 80% Ethanol
- A63880, Agencourt AMPure XP 5 ml

Safety warnings

- Do not use the kit past the expiration date.

Before start

- Check RNA sample quality prior to library preparation. RNA sample quality may vary between sample preparations.
- DTT in buffers may precipitate after freezing. If precipitate is seen, vortex buffer for 1-2 minutes or until the precipitate is in solution. The performance of the buffer is not affected once precipitate is in solution.
- Ensure pipettes are properly calibrated as library preparations are highly sensitive to pipetting error.
- Do not heat the RNA-Seq Adapters above room temperature.
- Vortex and micro-centrifuge each component prior to use, to ensure material has not lodged in the cap or the side of the tube.

STEP A: RNA Fragmentation

1 Adjust RNA volume to 14 µL by nuclease-free water.

2 For each reaction combine the following in a nuclease-free microcentrifuge tube or plate:

14 µL	RNA (in Nuclease-free Water or Elution Buffer)
5 µL	NEXTflex™ RNA Fragmentation buffer
19 µL	TOTAL

3 Mix thoroughly by pipetting.

4 Heat for 7-10 minutes at 95°C, immediately place on ice.
(incubation time based on post depletion QC-quality results choose from 7-10min or else)

5 Proceed to Step B: First Strand Synthesis.

STEP B: First Strand Synthesis

10m

6 Note: Due to the viscosity of certain materials, attempting to prepare more than the stated number of reactions may result in a shortage of materials. All NEXTflex enzyme components must be centrifuged at 600xg for 5 seconds before opening the tube(s).
For each reaction, add 1 µL NEXTflex First Strand Synthesis Primer to the fragmented RNA (from Step A).

7 Incubate at 65°C for 5 minutes, and immediately place on ice.

8 For each reaction, combine the following in a nuclease-free microcentrifuge tube or plate:

20 µL	Fragmented RNA + NEXTflex™ First Strand Synthesis Primer
4 µL	NEXTflex™ Directional First Strand Synthesis Buffer Mix
1 µL	NEXTflex™ Rapid Reverse Transcriptase
25 µL	TOTAL

9 Mix thoroughly by pipetting.

10 Incubate at:

25 °C 10 min

50 °C 50 min

70 °C 10 min

Proceed to Step C: Second Strand Synthesis

STEP C: Second Strand Synthesis

11 For each reaction combine the following in a nuclease-free microcentrifuge tube or plate:

25 µL	First Strand Synthesis product (from Step B)
25 µL	NEXTflex™ Directional Second Strand Synthesis Mix (contains dUTP)
50 µL	TOTAL

12 Mix thoroughly by pipetting

13 Incubate 16 °C 60 min .

Proceed to Step D: Bead Cleanup.

STEP D: Bead Cleanup

14 (following manufacturer's protocol for beads clean up)

1. Add 90 µL of well mixed AMPure XP Beads to each sample. Mix thoroughly by pipetting.
2. Incubate the tube/plate for 5 minutes at room temperature.
3. Place the tube/plate on the magnetic stand for 5 minutes at room temperature or until the supernatant appears completely clear.

4. Remove and discard all of the supernatant from the tube/plate taking care not to disturb the beads.
5. With the tube/plate on stand, add 200 µL of freshly prepared 80% ethanol to each well without disturbing the beads and incubate the tube/plate for at least 30 seconds at room temperature. Carefully, remove and discard the supernatant.
6. Repeat step 5, for a total of two ethanol washes. Ensure the ethanol has been removed.
7. Remove the tube/plate from the magnetic stand and let dry at room temperature for 2 minutes.
8. Resuspend dried beads in 17 µL of Resuspension Buffer. Mix thoroughly by pipetting. Ensure that the beads are completely rehydrated and resuspended.
9. Incubate resuspended beads at room temperature for 2 minutes.
10. Place the tube/plate on the magnetic stand for 5 minutes at room temperature or until the supernatant appears completely clear.
11. Transfer 16 µL of the clear supernatant to a fresh well for the next step.
12. The procedure may be stopped at this point and the reactions stored at -20°C.

STEP E: Adenylation

15 For each sample, combine the following reagents on ice in a nuclease-free pcr tube:

16 µL	Second strand synthesis DNA (from Step D)
4.5 µL	NEXTflex™ Adenylation Mix
20.5 µL	TOTAL

16 Mix thoroughly by pipetting.

17 Incubate at:

37 °C 30min

70 °C 5min

18 Proceed to Step F: Adapter Ligation.

STEP F: Adapter Ligation

19 For each sample, combine the following reagents on ice in a nuclease-free 96 well PCR Plate:

20.5 µL	3' Adenylated DNA (from Step E)
27.5 µL	NEXTflex™ Ligation Mix
2.0 µL	NEXTflex™ RNA-Seq Barcode Adapter 1 or RNA-Seq Barcode
50 µL	TOTAL

20 Mix thoroughly by pipetting.

21 Incubate on a thermocycler for 10 minutes at 30°C.

22 Proceed to Step G: Bead Cleanup.

STEP G: Bead Cleanup

23 (following manufacturer's protocol for beads clean up)
(increase final elution amount of resuspension buffer from 36 µL to 37 µL for downstream post adaptor Qubit assay)

1. Add 50 µL of well mixed AMPure XP Beads to each well containing sample. Mix thoroughly by pipetting.
2. Incubate the plate for 5 minutes at room temperature.
3. Place the plate on the magnetic stand for 5 minutes at room temperature or until the supernatant appears completely clear.
4. Remove and discard all of the supernatant from the plate taking care not to disturb the beads.
5. With the plate on the magnetic stand, add 200 µL of freshly prepared 80% ethanol to each well without disturbing the beads and incubate the plate for at least 30 seconds at room temperature. Carefully, remove and discard the supernatant.
6. Repeat step 5, for a total of two ethanol washes. Ensure the ethanol has been removed.
7. Remove the plate from the magnetic stand and let dry at room temperature for 2 minutes or until bead pellet is dry.
8. Resuspend dried beads in 51 µL of Resuspension Buffer. Mix thoroughly by pipetting. Ensure that the beads are completely rehydrated and resuspended.
9. Incubate resuspended beads at room temperature for 2 minutes.
10. Place the plate on the magnetic stand for 5 minutes at room temperature or until the supernatant appears completely clear.
11. Transfer 50 µL of the clear supernatant to a fresh well.

12. Add 50 µL of well mixed AMPure XP Beads to each well containing sample. Mix thoroughly by pipetting.
13. Incubate the plate for 5 minutes at room temperature.
14. Place the plate on the magnetic stand for 5 minutes at room temperature or until the supernatant appears completely clear.
15. Remove and discard all of the supernatant from the plate taking care not to disturb the beads.
16. With the plate on the magnetic stand, add 200 µL of freshly prepared 80% ethanol to each well without disturbing the beads and incubate the plate for at least 30 seconds at room temperature. Carefully, remove and discard the supernatant.
17. Repeat step 16, for a total of two ethanol washes. Ensure the ethanol has been removed.
18. Remove the plate from the magnetic stand and let dry at room temperature for 2 minutes or until the bead pellet is dry.
19. Resuspend the dried beads in 37 µL of Resuspension Buffer. Mix thoroughly by pipetting. Ensure that the beads are completely rehydrated and resuspended. (take 1µl from the samples while on the magnet rack for qubit assay)
20. Incubate resuspended beads at room temperature for 2 minutes.
21. Place the plate on the magnetic stand for 5 minutes at room temperature or until the supernatant appears completely clear.
22. Transfer 35 µL of the clear supernatant to a fresh well for the next step.
23. The procedure may be stopped at this point and the reactions stored at -20°C.

(additional step) post adapter Qubit

24 For determining the ideal PCR amplification cycle numbers to be used in the next step, a Qubit assay was performed after adaptor ligation. We used the Qubit dsDNA HS assay kit (0.2 – 100 ng) following manufacturer's protocol.

STEP H: PCR Amplification

25 For each sample, combine the following reagents on ice in a pcr tube:

35 µL	Adapter Ligated DNA
1 µL	NEXTflex™ Uracil DNA Glycosylase
12 µL	NEXTflex™ PCR Master Mix
2 µL	NEXTflex™ Primer Mix
50 µL	TOTAL

26 Mix thoroughly by pipetting.

27 For the PCR amplification, the following cycling conditions were used:

30 min	37°C	
2 min	98°C	
30 sec	98°C	Repeat 15 cycles*
30 sec	65°C	
60 sec	72°C	
4 min	72°C	

*PCR cycles will vary depending on the amount of starting material and quality of your sample. Further optimization may be necessary. Always use the minimum number of cycles possible. (determine the cycle number according to the results of Qubit)

28 Proceed to Step I: Bead Cleanup.

STEP I: Bead Cleanup

29 (following manufacturer's protocol for beads clean up)

1. Add 40 µL of well mixed AMPure XP Beads to each well containing sample. Mix thoroughly by pipetting.
2. Incubate the plate for 5 minutes at room temperature.
3. Place the plate on the magnetic stand for 5 minutes at room temperature or until the supernatant appears completely clear.
4. Remove and discard all of the supernatant from the plate taking care not to disturb the beads.
5. With plate on stand, add 200 µL of freshly prepared 80% ethanol to each well without disturbing the beads and incubate the plate for at least 30 seconds at room temperature. Carefully, remove and discard the supernatant.
6. Repeat step 5, for a total of two ethanol washes. Ensure the ethanol has been removed.
7. Remove the plate from the magnetic stand and let dry at room temperature for 2 minutes or until the bead pellet is dry.
8. Resuspend dried beads in 51 µL of Resuspension Buffer. Mix thoroughly by pipetting. Ensure that the beads are completely rehydrated and resuspended.

9. Incubate resuspended beads at room temperature for 2 minutes.
10. Place the plate on the magnetic stand for 5 minutes at room temperature or until the supernatant appears completely clear.
11. Transfer 50 µL of the clear supernatant to a fresh well.
12. Add 40 µL of well mixed AMPure XP Beads to each well containing sample. Mix thoroughly by pipetting.
13. Incubate the plate for 5 minutes at room temperature.
14. Place the plate on the magnetic stand for 5 minutes at room temperature or until the supernatant appears completely clear.
15. Remove and discard all of the supernatant from the plate taking care not to disturb the beads.
16. With the plate on the magnetic stand, add 200 µL of freshly prepared 80% ethanol to each well without disturbing the beads and incubate the plate for at least 30 seconds at room temperature. Carefully, remove and discard the supernatant.
17. Repeat step 16, for a total of two ethanol washes. Ensure the ethanol has been removed.
18. Remove the plate from the magnetic stand and let dry at room temperature for 2 minutes or until the bead pellet is dry.
19. Resuspend dried beads in 32 µL of Resuspension Buffer. Mix thoroughly by pipetting. Ensure that the beads are completely rehydrated and resuspended. (take 1µl from the samples while on the magnet rack for qPCR dilution)
20. Incubate resuspended beads at room temperature for 2 minutes.
21. Place the plate on the magnetic stand for 5 minutes at room temperature or until the supernatant appears completely clear.
22. Transfer 30 µL of the clear supernatant to a fresh well.
23. We recommend quantifying your library with a fluorometer and checking the size using an Agilent Bioanalyzer. If on the Bioanalyzer trace there are two bands, one of expected size and one of higher molecular weight, a portion of your adapter ligated inserts have annealed to each other forming a bubble product. This occurs due to the long adapter length and is more prevalent when there are too many PCR cycles. This type of double band will not affect your sequencing results as the double stranded product will be denatured prior to cluster generation. As an extra verification step, a portion of your product can be denatured manually by heating the sample to 95°C for 5 minutes and then placing it on ice. The denatured product should appear as a single band on a Bioanalyzer RNA Pico 6000 Chip Kit.
24. qPCR is recommended to quantitate DNA library templates for optimal cluster density. This can be performed using any qPCR quantification kit with the NEXTflex Primer Mix.
25. Non-multiplexed libraries can be normalized using Tris-HCl (10 mM), pH 8.5 with 0.1% Tween 20. For multiplexed libraries, transfer 10 µL of each normalized library for pooling in the well of a new 96 Well PCR Plate. Mix thoroughly by pipetting.

30 The library is now ready for cluster generation with the standard Illumina protocol. Proceed to cluster generation or store at -20°C.
