# Supplementary material for: Evaluation of metatranscriptomic sequencing protocols to obtain full-length RNA virus genomes from mammalian tissues
Source: PLoS One. 2025 May 30;20(5):e0324537. doi: 10.1371/journal.pone.0324537 (PMC12124746; doi:10.1371/journal.pone.0324537)
Supplement: S5 File — Also available on protocols.io: https://dx.doi.org/10.17504/protocols.io.kqdg3p6pel25/v1 (PDF) [file pone.0324537.s005.pdf]

Jul 18, 2022

NGS workflow with rRNA depletion for viral RNA sequencing from animal tissue specimens

DOI
dx.doi.org/10.17504/protocols.io.kqdg3p6pel25/v1

Yiqiao Li[1], Magda Bletsa[2], Ine Boonen[1], Philippe Lemey[1]

[1]Department of Microbiology, Immunology and Transplantation, Rega Institute, KU Leuven – University of Leuven, Leuven, Belgium;
[2]National and Kapodistrian University of Athens

KU Leuven

Yiqiao Li
Ruijin Hospital, Shanghai Jiao Tong University School of Med...

OPEN ACCESS

DOI: dx.doi.org/10.17504/protocols.io.kqdg3p6pel25/v1

Protocol Citation: Yiqiao Li, Magda Bletsa, Ine Boonen, Philippe Lemey 2022. NGS workflow with rRNA depletion for viral RNA sequencing from animal tissue specimens . **protocols.io** **https://dx.doi.org/10.17504/protocols.io.kqdg3p6pel25/v1**

License: This is an open access protocol distributed under the terms of the **Creative Commons Attribution License**, which permits unrestricted use, distribution, and reproduction in any medium, provided the original author and source are credited

Protocol status: Working
We use this protocol and it's working with NGS library preparation aiming to generate complete hepacivirus genomes from animal samples.

Created: January 19, 2022

Last Modified: July 18, 2022

Protocol Integer ID: 57120

Disclaimer

DISCLAIMER – FOR INFORMATIONAL PURPOSES ONLY; USE AT YOUR OWN RISK

The protocol content here is for informational purposes only and does not constitute legal, medical, clinical, or safety advice, or otherwise; content added to **protocols.io** is not peer reviewed and may not have undergone a formal approval of any kind. Information presented in this protocol should not substitute for independent professional judgment, advice, diagnosis, or treatment. Any action you take or refrain from taking using or relying upon the information presented here is strictly at your own risk. You agree that neither the Company nor any of the authors, contributors, administrators, or anyone else associated with **protocols.io**, can be held responsible for your use of the information contained in or linked to this protocol or any of our Sites/Apps and Services.

Abstract

This NGS workflow describes how to prepare libraries from total RNA with a rRNA depletion step to increase the yield of non-host RNA transcripts.
This workflow was initially designed with the aim of generating full-length hepaciviruses, but can also be used for sequencing any other RNA viral sequences or RNA-related metagenomes.

Materials

A	B	C
KK4601	KAPA SYBR® FAST Master Mix (2X) Universal	Roche
Q10210	Qubit RNA BR assay kit (100 assays)	Thermo fisher
5067-1511	Agilent RNA 6000 Nano kit (reagents + chips)	Agilent
E6310 (S/L/X)	NEBNext rRNA Depletion kit (Human/Mouse/Rat)	NEB
A63987	NEBNext RNA sample purification beads, Agencourt RNA clean XP beads from Beckman coulter	Beckman Coulter
NOVA-5138-08, 48 rxns	NextFlex Rapid directional RNAseq kit	PerkinElmer
Cat # 512911, 512912, 512913, 512914	NEXTflex RNA-Seq Barcodes – 6 /12/ 24 / 48	PerkinElmer
A63880	Agencourt AMPure XP 5 ml	Beckman Coulter
Q32851 (100 assays), Q32854 (500 assays)	Qubit dsDNA HS assay kit	Thermofisher
Q32856 (500 tubes)	Qubit Assay tubes	Thermofisher
5067-4626	Bioanalyzer Agilent High Sensitivity DNA Kit (110 samples) (chips and reagents)	Agilent
5067-4627	Bioanalyzer Agilent High Sensitivity DNA reagents (enough for 10 chips)	Agilent
7960336001	KK4873, complete kit Rox Low KAPA Library Quanitification Kits - Complete Kit (ROX Low)	Kapa biosystems
	TET-buffer (Tris-Cl (10mM) EDTA (1mM) Tween 20 (0.1%)	
Library amplification		
PCR Primer 1	AATGATACGGCGACCACCGAGATCTACAC	
PCR Primer 2	CAAGCAGAAGACGGCATACGAGAT	

Safety warnings

To minimise the effect of index hopping, libraries need to be stored individually at -20 °C. Prepare the pools only when you are ready to send those for sequencing.

1. Pre quantity QC

1 The quantity of total RNA is evaluated using Qubit RNA BR assay kit (following manufacturer's protocol) 30m

2. Pre quality QC

2 The quality of total RNA is evaluated using the Agilent RNA 6000 Nano kit in Bioanalyzer (following the manufacturer's protocol)
(based on previous Qubit results and the bioanalyzer kit range, prepare samples dilution 1:10 or else for bioanalyzer)
Samples with RIN>2 can proceed to the next step of rRNA depletion 1h

3. rRNA depletion

3 Adjust your starting material, which can be anything between 5 ng–1 µg total RNA (DNA free), according to the Qubit results in a 12 µl total volume reaction with nuclease-free water, and follow the manufacturer's manual of NEBNext rRNA Depletion kit (Human/Mouse/Rat) to remove total rRNA.
This kit removes any rRNA while retaining the viral RNA. Upon incubation, we evaluate our results using a custom qPCR assay targeting the 12S rRNA and the NS3 genomic region of hepaciviruses.
Analogous evaluation is recommended while working with other viruses. 3h

Protocol

NAME
NEBNext rRNA Depletion Kit (Human/Mouse/Rat) E6310

CREATED BY
Isabel Gautreau

PREVIEW

4. Post quantity/quality QC

4 Upon rRNA depletion, the RNA product is quantified and qualified using the Qubit RNA BR assay kit (or Qubit RNA HS assay kit based on the results) and the Agilent RNA 6000 Pico kit following manufacturer's protocol. 1h 30m

5. Library preparation

5 Based on the Qubit results obtained in step 4, set the total amount of rRNA-depleted RNA input to anything between ~1ng - 100 ng.

6 Adjust to 14µL with nuclease-free water.

7 Follow the protocol of NGS library preparation using the NEXTFLEX Rapid Directional RNAseq kit (NOVA-5138-08) for tissue samples.
(will be released soon, current private link: https://www.protocols.io/private/46D9A030792211ECB5450A58A9FEAC02)

5. Library quality/quantity QC

8 Check the fragment size of your libraries using Bioanalyzer with the Agilent High Sensitivity DNA kit
(following manufacturer's protocol, starting material: 1 µL)
The ideal fragment size for Illumina sequencing should range between 350-500bp.

9 Check the total amount of prepared libraries for pooling and confirm whether the adaptors are well ligated. This can be tested using a qPCR assay for each library with the complete Rox Low kit with Illumina general primers (catalogue number KK4873).
Prepare dilutions of your DNA libraries in 1:10, 1:1000, and 1:8000 with TET-buffer/nuclease-free water.
Prepare the master mix as follows:
KAPA Master Mix 12 µl
H2O 4 µl
Diluted DNA 4 µl
(total volume 20 µl)

Load in 96-well plate
The PCR cycling conditions are listed below :

Step 1	Initial denaturation	95°C	5'	
Step 2	Denaturation	95°C	30''	35x
Step 3	Annealing/extension	60°C	45''	
Step 4	Dissociation	As proposed by the software		

6. Library pooling

10 Pool libraries according to the library requirements of your sequencing platform.
Pool together 8-12 libraries (differs according to customers' demands for deep or ultra-deep sequencing) and adjust the volume using the same reagent as the one used for the elution in the library preparation step.
Ready for sequencing!
